# Persistence of Pathogens with Short Infectious Periods in Seasonal Tick Populations: The Relative Importance of Three Transmission Routes

**DOI:** 10.1371/journal.pone.0011745

**Published:** 2010-07-23

**Authors:** Etsuko Nonaka, Gregory D. Ebel, Helen J. Wearing

**Affiliations:** 1 Department of Biology and Department of Mathematics and Statistics, University of New Mexico, Albuquerque, New Mexico, United States of America; 2 Department of Pathology, University of New Mexico Medical School, Albuquerque, New Mexico, United States of America; University of Liverpool, United Kingdom

## Abstract

**Background:**

The flaviviruses causing tick-borne encephalitis (TBE) persist at low but consistent levels in tick populations, despite short infectious periods in their mammalian hosts and transmission periods constrained by distinctly seasonal tick life cycles. In addition to systemic and vertical transmission, cofeeding transmission has been proposed as an important route for the persistence of TBE-causing viruses. Because cofeeding transmission requires ticks to feed simultaneously, the timing of tick activity may be critical to pathogen persistence. Existing models of tick-borne diseases do not incorporate all transmission routes and tick seasonality. Our aim is to evaluate the influence of seasonality on the relative importance of different transmission routes by using a comprehensive mathematical model.

**Methodology/Principal Findings:**

We developed a stage-structured population model that includes tick seasonality and evaluated the relative importance of the transmission routes for pathogens with short infectious periods, in particular Powassan virus (POWV) and the related “deer tick virus,” emergent encephalitis-causing flaviviruses in North America. We used the next generation matrix method to calculate the basic reproductive ratio and performed elasticity analyses. We confirmed that cofeeding transmission is critically important for such pathogens to persist in seasonal tick populations over the reasonable range of parameter values. At higher but still plausible rates of vertical transmission, our model suggests that vertical transmission can strongly enhance pathogen prevalence when it operates in combination with cofeeding transmission.

**Conclusions/Significance:**

Our results demonstrate that the consistent prevalence of POWV observed in tick populations could be maintained by a combination of low vertical, intermediate cofeeding and high systemic transmission rates. When vertical transmission is weak, nymphal ticks support integral parts of the transmission cycle that are critical for maintaining the pathogen. We also extended the model to pathogens that cause chronic infections in hosts and found that cofeeding transmission could contribute to elevating prevalence even in these systems. Therefore, the common assumption that cofeeding transmission is not relevant in models of chronic host infection, such as Lyme disease, could lead to underestimating pathogen prevalence.

## Introduction

Tick-borne encephalitis virus (TBEV) causes thousands of human cases of tick-borne encephalitis (TBE) in Europe and Asia every year [1], [2], [3]. Recently, reports of TBE due to infection with other members of the tick-borne encephalitis serological complex – Powassan virus and the related “deer tick virus” [both referred to hereafter as Powassan virus (POWV)] – have been increasing in the north-eastern and north central United States and south-eastern Canada [4], [5]. This group of encephalitis-causing viruses belongs to the genus Flavivirus and infection can result in severe morbidity, long-term neurological sequelae, and possible death [6]–[8]. Although many fewer human infections by tick-borne flaviviruses are reported in North America than Europe (a single digit vs. thousands) [2], [4], increasing incidence in the former suggests the potential for an emergent disease in humans [4], [9]. In fact, the reported prevalence levels of POWV in adult tick populations in the United States compare with those of TBEV in Europe (>0–5%) [10]–[12]. Understanding the mechanisms driving POWV transmission, and ultimately its prevalence in ticks and risk to human health, requires a comprehensive examination of the complex interactions among the pathogen, the tick vector, and the host populations.

Several mechanisms may promote or hinder persistence of POWV in North America. Tick-borne flaviviruses typically have short infectious periods (about 2–3 days) in mammalian hosts, limiting direct transmission from host to tick. Therefore, POWV infection of ticks by systemic transmission (transmission between host and tick via feeding on a viraemic, infectious host) seems much less efficient than for pathogens with longer or chronic infections such as *Borrelia burgdorferi*, the causative agent of Lyme disease. Alternatively, a different form of horizontal transmission – cofeeding transmission (or saliva-activated transmission) [13]–[16] between infected and uninfected ticks feeding in close proximity on non-viraemic, including immune, hosts – has been proposed as the key pathway that can sustain TBEV in ticks [11], [15]. Cofeeding transmission is potentially effective because ticks show aggregated distributions among host individuals such that 75% of ticks feed on 20% of hosts [17]. However, because (a) cofeeding transmission requires ticks to feed in close proximity on the same host and (b) tick activity is seasonal in temperate regions [18]–[20], the timing of tick activity may be critical to the persistence of tick-borne flaviviruses. In addition, although limited evidence suggests that vertical transmission (transmission from adult female ticks to their eggs) is relatively inefficient (0.1%) [21]–[23], when coupled with the high fecundity of ticks (∼2000 eggs per female), vertical transmission could significantly increase prevalence and/or support virus persistence in nature.

Evaluating the relative contribution of each mode of transmission to POWV persistence under the constraints of tick seasonality requires a theoretical framework that incorporates all potential mechanisms. However, previous models of tick-borne pathogens have investigated only subsets of the important factors outlined above. Tick seasonal life cycles have been incorporated into Lyme disease models [24]–[26] but rarely into TBEV models [27]. Most Lyme disease models do not include cofeeding transmission because the infectious period in the host is so long that systemic transmission is assumed to serve as the major pathway. Rosà and colleagues [28], [29] have developed models for TBEV including cofeeding transmission but did not include tick seasonality, which is a distinct feature of the sheep tick (*Ixodes ricinus*) in Europe as well as the black-legged tick (*Ixodes scapularis*), an emergent vector of POWV in North America [9]. Furthermore, existing models do not examine the interaction of cofeeding and vertical transmission. Ogden et al. [27] developed a detailed model of tick-borne pathogen transmission in seasonal tick populations with systemic and cofeeding transmission, but failed to include vertical transmission. Randolph and colleagues have proposed that cofeeding transmission between larvae and nymphs (inter-cohort cofeeding transmission) is critically important and vertical transmission has an insignificant role in TBEV persistence in the sheep tick [17], [23], [30]. If vertical transmission is not important, overlap between cohorts of ticks must provide the critical link for the pathogen to be passed from one generation to the next via horizontal transmission. Hartemink et al. [31] evaluated this theory using a non-dynamic model, but a study that explicitly considers seasonality in the tick life cycle could directly assess the effects of inter-cohort transmission on the prevalence and persistence of TBEV.

In this paper, we constructed and analyzed a comprehensive stage-structured population model to understand POWV persistence in seasonal *I. scapularis* populations. In addition to evaluating the relative importance of systemic, cofeeding and vertical transmission, we examined the effects of inter-cohort overlap on pathogen persistence and prevalence. Our analyses were based on both numerical simulations and the next generation matrix method, which was used to calculate R_0_ and to assess the sensitivity of R_0_ to changes in the values of model parameters [31]–[33]. Through this work, we shed some light on the mechanisms for relatively low but consistent prevalence of POWV and TBEV, vectored by closely related Ixodid tick species, observed in the United States [10], [12] and in Europe [11], respectively.

## Materials and Methods

### Tick life cycle

**Figure 1:**
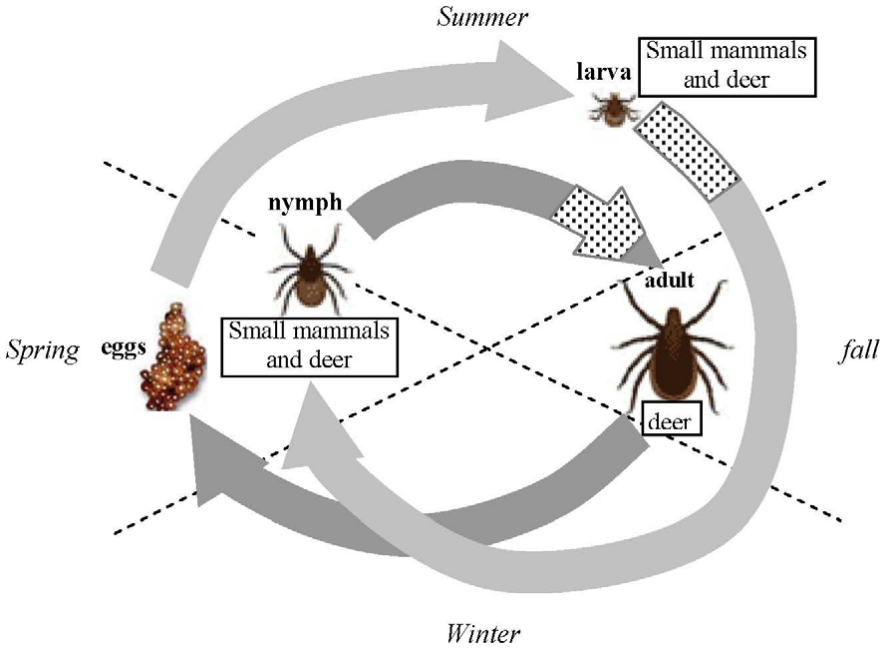
The typical life cycle of the black-legged or deer tick (*Ixodes scapularis*). The light grey arrows represent the first year, and the dark grey arrows indicate the second year. The dotted areas on the arrows indicate the time period where two cohorts can overlap. Larvae emerge in mid to late summer and quest for 3–4 months and then moult followed by diapause in fall until next spring. The nymphs emerge in late spring and quest for 3–4 months before they moult into adults in fall. The adults emerge in fall and quest until late spring except during the coldest months when they undergo diapause. The larvae and nymphs usually feed on small mammals, mainly rodents, for blood meals, while the adults feed on larger mammals such as deer. The figure is based on Fig. 1 in [34]. Temporal dynamics of the tick and host populations from a typical run of the model is presented in Fig. S1.

We mainly focus on the ecology of the transmission cycle of POWV involving *I. scapularis* in upper mid-western and north-eastern regions of the United States, where ticks typically have a two year life cycle (Fig. 1). The timings for emergence and the onset of diapause may vary geographically and from year to year [34]. For simplicity, we assume an average condition. In our model, the larvae are active from July to October, the nymphs from May to August, and the adults from October to May except January and February. Ticks feed only once in each stage. The most common rodent host species is the white-footed mouse (*Peromyscus leucopus*) which is treated as competent hosts (H_1_) in our model [35]. The white-tailed deer (*Odocoileus virginianus*) is abundant in the regions but considered incompetent in our model (H_2_). Once infected, infection in ticks is life long, while it lasts only a few days in the competent hosts.

### The model

We transformed and extended the continuous-time model developed by Rosà and Pugliese [29] for TBEV into a discrete-time model to include the seasonal life cycle of the tick, using the multiple matrix model approach introduced by Sandberg et al. [26] for Lyme disease. The model by Rosà and Pugliese [29] was modified to also include the three transmission routes (vertical, cofeeding, and systemic). Our model is composed of monthly matrices representing population processes occurring in each month for different “types” of ticks and for the host species (a more detailed description of the model and the equations are available in Supporting Information S1). The model computes density (per ha) for twelve types of ticks; three life stages (larvae, nymphs, adults), two feeding phases (fed or unfed), and two epidemiological conditions (susceptible or infected). If ticks fail to feed during the questing season, they are assumed to die from starvation. For the hosts, we assume no seasonal life cycle and a constant total population size. This is an over-simplification but we are focused on the effects of tick seasonality in this paper. For the competent host (able to carry and transmit the pathogen, denoted as H_1_), the density of susceptible, infected, or recovered types changes over time as hosts become infected and recover from the infection. To accommodate the time scales of the host infectious period (2–3 days), we approximate the monthly host dynamics using discrete-time equations derived from the integration of differential equations that describe the within-month infection, recovery, and mortality processes in the host population. The competent host is born uninfected, and infection only occurs after birth. The incompetent host (H_2_) does not develop viraemia and is represented as one constant state variable. We assume that infection with the pathogen does not affect feeding rates of ticks or demographic characteristics in ticks or hosts. Temporal dynamics of the populations from a representative simulation run is shown in Fig. S1.

We parameterized the model with values taken from the literature (Table 1, Table S1 for literature sources). If available, we utilized studies with *I. scapularis*, but otherwise we used values for a closely related and widely studied European tick, *I. ricinus*. Parameter values related to pathogen transmission are mostly from laboratory studies, while many demographic parameters are estimated in field studies. Because transmission rates were measured in laboratory conditions, it is uncertain how well these values represent field conditions. To reflect this uncertainty, we vary the three transmission rates over plausible ranges based on current estimates. For cofeeding transmission, we considered the measured values as theoretical maxima (0.24 on recovered hosts and 0.72 on susceptible or infected hosts) due to the unnatural settings of the experiments [14]. The two cofeeding transmission rates – depending on the immunity status of the competent host – are varied simultaneously by a tuning multiplier (between 0 and 1) and are expressed in the figures as fractions of the measured values (e.g., 0.5 on the axis corresponds to 0.12 for cofeeding transmission on recovered hosts and 0.36 on susceptible or infected hosts, before adjustments by aggregation factors). For vertical transmission, we used the value referred to in Danielová et al. [22] and also estimated a maximum possible value from the limited field data presented in their paper (4–6 larvae out of 419 larvae caught— 1–1.4% — were potentially infected before their first blood meal). Although the current accepted estimate of vertical transmission is 0.1%, there is some evidence from [22] supporting higher values, and so we varied the rate up to 2% in the simulations.

**Table 1:** Parameter definitions and the values used in the simulations.

Parameter	Definition	Value
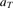	Number of eggs laid per female tick	2000
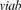	Fraction of eggs that produce viable active larvae	0.4
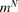	Probability of female nymph-to-adult molting	0.5·exp(−0.09)
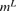	Probability of larva-to-nymph molting	exp(−0.9)
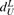	Mortality rate of unfed larvae	−log((1−0.0067)^30^)
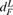	Mortality rate of fed larvae	−log((1−0.00208)^30^)
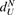	Mortality rate of unfed nymphs	−log((1−0.0021)^30^)
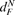	Mortality rate of fed nymphs	−log((1−0.0007)^30^)
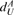	Mortality rate of unfed adults	−log((1−0.00095)^30^)
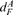	Mortality rate of fed adults	−log((1−0.0006)^30^)
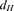	Survival probability of host 1 (H_1_)	exp(−0.25)
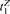	Number of ticks feeding on a H_1_ individual	27.8 (L), 5 (N), 0 (A)
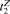	Number of ticks feeding on a H_2_ individual	239 (L), 20 (N), 30 (A)
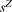	Number of questing days per month	30 (L, N, A)
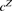	Number of days ticks remain attached on a host	3 (L), 5 (N), 10 (A)
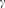	Recovery rate of H_1_	0.3·(30)
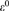	Probability of vertical transmission	0.001* [0,0.02]
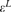	Probability of larva-nymph trans-stadial transmission	0.22
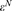	Probability of nymph-adult trans-stadial transmission	0.54
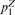	Probability of H_1_-to-tick systemic transmission	0.9* [0,0.9] (L, N)
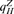	Probability of tick-to-H_1_ systemic transmission	0.8 (L, N), 0 (A)
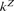	Aggregation parameter	1.19 (L), 0.56 (N)
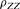	Correlation coefficient (LL:larva-larva, NN: nymph-nymph, LN: larva-nymph)	1 (LL, NN), 0.2 (LN)
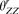	Probability of cofeeding transmission on a recovered H_1_ (LL: from a larva to a larva, LN: from a larva to a nymph, etc)	0.24* [0,0.24] (all)
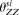	Probability of cofeeding transmission on a susceptible or infected H_1_	0.72* [0,0.72] (all)

The superscript Z specifies age class (L, N, or A). All the rates are per month. Parameters with an asterisk (*) were varied in simulations (the ranges in brackets). Sources from which the values are taken are listed in the extended version of this table in Table S1.

In our model, the feeding rate of the tick and the rate of cofeeding transmission depend on the densities of ticks and hosts (and are therefore updated monthly). Encounters (and feeding) between questing ticks and either species of hosts are modelled as mass action [26], [29]. The calculation of cofeeding transmission rates incorporates the effects of tick aggregation and reflects the distribution of tick load amongst host individuals that has been described as negative binomial [11], [23]. The infection rate of the competent host is also modelled as mass action [29]. We assume that adult ticks do not feed on the competent hosts since the majority of adult ticks feed on large mammals (i.e., 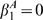) [36]–[38]. Infected host individuals can recover and develop immunity to the pathogen. The host does not infect other host individuals (no direct horizontal or vertical transmission within the host population). We simulate the model until the tick population reaches a stable annual density cycle.

### Next generation matrix and R_0_

To compute R_0_ we utilized the next-generation matrix method [31]–[33], [39]. Hartemink et al. [31] and Matser et al. [33] applied it to tick-borne diseases, and we closely follow their steps except that we obtain the expected numbers of new infections (i.e., the elements of the matrix) directly from model simulations. Briefly, the next generation matrix contains the numbers of individuals that are infected by one infected individual of each type-at-birth during the time it is infectious. Types-at-birth refer, in our model, to ticks or H_1_ hosts that become infected in one of the months they can become infected (“birth” of an infected). We label the types-at-birth as 1) ticks infected as an egg (via vertical transmission; row 1), 2) ticks infected as larvae (through their first blood meal; rows 2-5, August–November), 3) ticks infected as nymphs (through their second blood meal; rows 6–9, June–September), and 4) systemically infectious competent hosts (rows 10–15, June–November). The types-at-birth of *infecting* individuals are the columns, and those of *infected* individuals are the rows (Table S2). For example, a larva infected in August (L8) could grow into an infected nymph (since larvae are infected upon feeding, the next opportunity to infect other individuals does not come until they feed next time as nymphs) and infect larvae in August or September, nymphs between June and September, H_1_ hosts between June and September, and/or lay infected eggs. Since the elements of the matrix represent pathogen transmission between particular pairs of types, we can classify the elements into the three transmission routes [31], [33]. The nonzero elements in the first row, for instance, represent the number of infections from ticks to eggs, hence vertical transmission. Cofeeding transmission corresponds to the elements representing infections directly from ticks to ticks. The dominant eigenvalue of this matrix can be interpreted as R_0_ with the property that, when R_0_>1, the disease can spread into a purely susceptible population [32].

To compute our next generation matrix, we introduce to the population at the “disease-free” stable-stage distribution one infected tick or host of one type-at-birth and calculate the number of newly infected individuals by type-at-birth. All newly infected individuals are immediately removed if the originally infected tick is to subsequently infect more secondary cases (to avoid including tertiary infections). We assumed that the infectiousness of ticks and hosts is independent of how they acquired the pathogen and distinguished 15 types-at-birth. For every month when ticks are actively searching a host, there is one type-at-birth for every tick life stage at which infection can be acquired. There is another type-at-birth for the competent host for each month they can be bitten by ticks (Table S2).

The next generation matrix, **K**, will be a 15×15 matrix. Each element of the matrix, k*_ij_*, indicates the expected number of secondary infections in type-at-birth *i* caused by one infected individual of type-at-birth *j* during its entire infectious period. Some of the elements are zero because not all types can infect all other types. Hosts cannot infect tick eggs and directly infect other hosts, for example. Since we assume adult ticks do not feed on the competent host, ticks infected during their second blood meal (i.e., nymphs) do not have an opportunity to infect other ticks except their own eggs. Because ticks infected during their first blood meal (i.e., larvae) feed and possibly infect other individuals only after they moult into nymphs, elements in columns 2–5 corresponding to the months when no nymphs feed are zero.

### Non-overlapping generations

To examine the effects of inter-cohort overlap (i.e., larvae and nymphs), we shift the feeding seasons of larvae (one month backward) and nymphs (one month forward) to remove the overlap between two generations. Since the types-at-births for the hosts increase by two, the next generation matrix becomes 17×17. The basic structure of the matrix is the same.

### Sensitivity and elasticity analyses

Another utility of the next generation matrix is the ease of computing sensitivity and elasticity values of the matrix elements and of the model parameters [31], [33], [40]. Sensitivities quantify how R_0_ changes in response to small changes in the value of a matrix element or a model parameter, while elasticities are normalized sensitivities and measure the proportional change in R_0_ in response to a proportional change in an element or a parameter. Elasticity analysis on the next generation matrix quantifies the relative importance of transmission routes with respect to R_0_ for a given set of parameter values. For matrix elements, the element-wise elasticities sum to unity and each elasticity value indicates the relative importance of a particular transmission route between two types-at-birth. Using this property, we can sum appropriate elements to calculate the relative importance of vertical, cofeeding, and systemic transmission. For model parameters, elasticity is a more convenient metric to assess sensitivity of R_0_ to a small change in a parameter because input values of parameters can vary by orders of magnitude. We examined the cases with and without cohort overlap for two sets of vertical and cofeeding transmission rates: lower vertical and higher cofeeding transmission (Case 1) and higher vertical and lower cofeeding transmission (Case 2). We chose the vertical and cofeeding transmission rates so that the prevalence is roughly the same (except in the non-overlapping case with low vertical transmission).

## Results

### The effect of the three transmission routes on pathogen prevalence and R_0_

**Figure 2:**
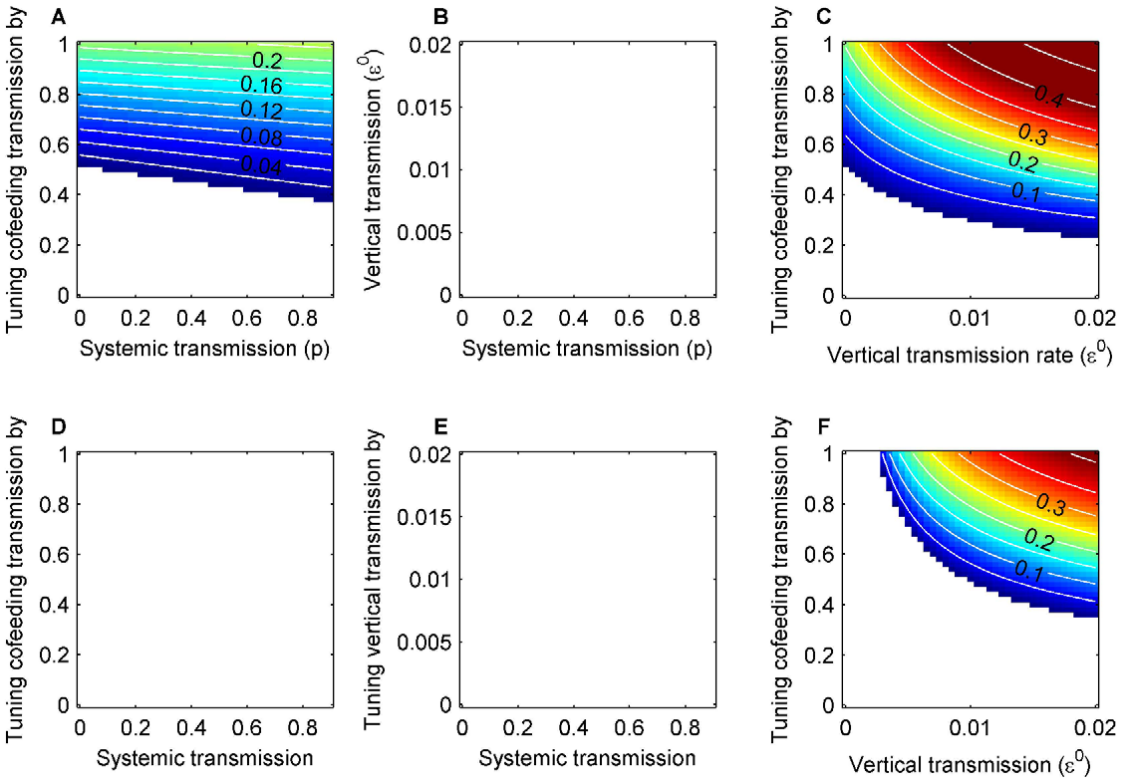
Pathogen prevalence in adult ticks in October with one route turned off at a time. The figures in the top row show the cases when the two cohorts (larvae and nymphs) overlap, and the bottom row show the cases when they do not overlap: without vertical transmission (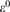 = 0; A, D), without cofeeding transmission (all 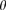
's = 0; B, E), and without systemic transmission (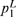 and 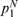 = 0; C, F). The blank colour means prevalence = 0. Cofeeding transmission is expressed as the multiples of the baseline rates (0.24 and 0.72). Neither vertical nor systemic transmission along can maintain the pathogen, while cofeeding transmission is necessary and can be sufficient by itself to sustain the pathogen in the tick population. The corresponding figures for R_0_ values are presented in Fig. S2.

The effect of each transmission route on prevalence and R_0_ was assessed by turning off one route at a time and varying the rates of the others over plausible ranges (Fig. 2, Fig. S2). The model outputs show that prevalence and R_0_ are qualitatively in close agreement. One source of discrepancy is that prevalence reflects endemic conditions (i.e., some hosts are immune), while all secondary infections are removed in the R_0_ calculation (i.e., all hosts are susceptible). In the absence of either vertical (Fig. 2A) or systemic transmission (Fig. 2C), the pathogen persists in the tick population at or above the observed level of >0–5%, while in the absence of cofeeding transmission the pathogen cannot be maintained (Fig. 2B). When cofeeding transmission is the only pathway (Fig. 2A, C at zero on the horizontal axes), the pathogen can still be maintained at intermediate or higher cofeeding transmission rates. Although the pathogen can be sustained without vertical transmission, a small amount can act in synergy with cofeeding transmission to boost prevalence (Fig. 2C). On the other hand, systemic transmission is of less importance (Fig. 2A). In summary, neither vertical nor systemic transmission alone can maintain the pathogen, but cofeeding transmission can. Cofeeding transmission is necessary and can be sufficient by itself to sustain the pathogen in the tick population.

When the two tick cohorts (i.e., larvae and nymphs) do not overlap, the pathogen does not persist without vertical or cofeeding transmission (Fig. 2D, E). Persistence is plausible only when both cofeeding and vertical transmission routes operate (Fig. 2F). Hence, overlap between cohorts has a large impact when there is no vertical transmission, whereas the effect is much less dramatic in the presence of both vertical and cofeeding transmission.

### Relative importance of the three transmission routes

**Figure 3:**
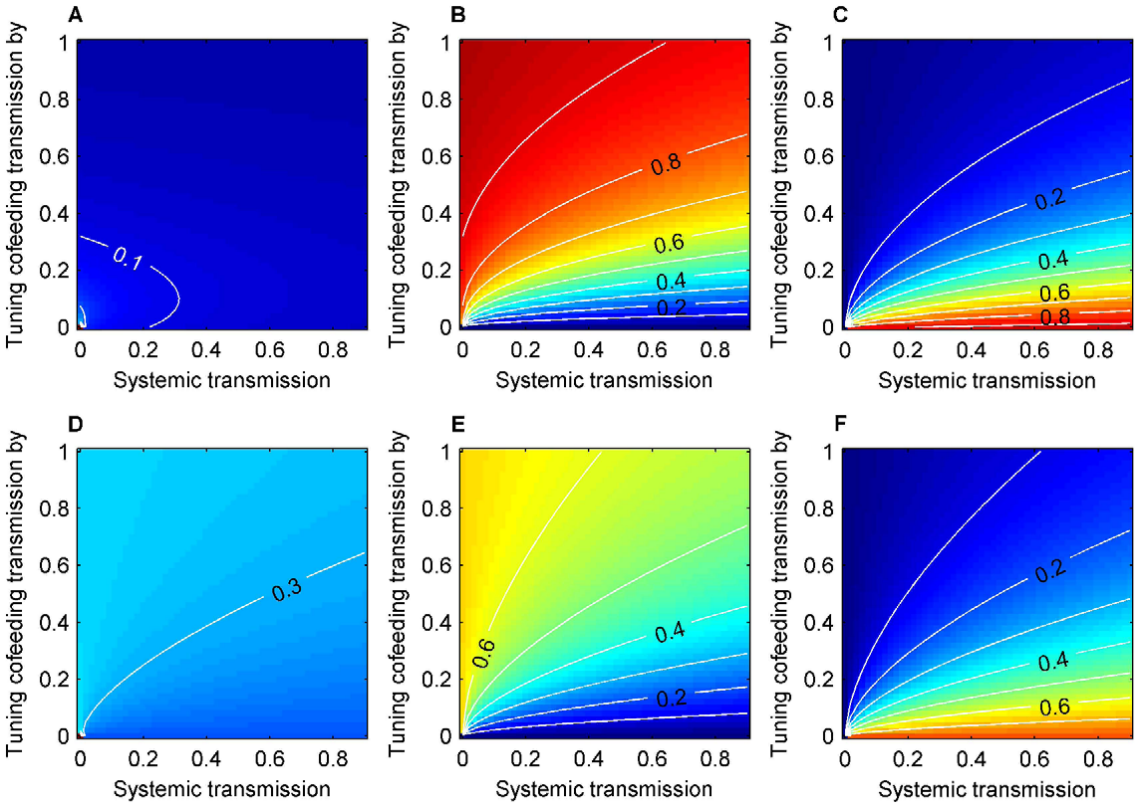
Relative importance of the three transmission routes. Elasticity values when two cohorts overlap for vertical (A, D), cofeeding (B, E), and systemic transmission (C,F) with the vertical transmission rate(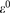) = 0.001, with inter-cohort overlap (A, B, C) and without (D, E, F). In both cases, relative importance of the three routes varies over the parameter space, notably cofeeding and systemic transmission exchanging relative importance as the baseline cofeeding transmission rate increases (Fig. 3B, C, E, F). Cohort overlap did not qualitatively change the results, but vertical transmission increased its relative importance by 10–15% (Fig. 3A,D).

Elasticity values can be interpreted as proportional contributions to R_0_ from transmission routes. We performed analysis with and without cohort overlap with vertical transmission at 0.1% (Fig. 3). In both cases, relative importance of the three routes varies over the parameter space, notably cofeeding and systemic transmission exchanging relative importance as the baseline cofeeding transmission rate increases (Fig. 3B, C, E, F). The relative importance of vertical transmission is less variable for the majority of the space (Fig. 3A, D). As expected, an increase in the vertical transmission rate increases its relative importance (Fig. S3, A). Cohort overlap did not qualitatively change the results, but vertical transmission increased its relative importance by 10–15% (Fig. 3A, D).

### Sensitivity and elasticity analyses for the input parameters

**Figure 4:**
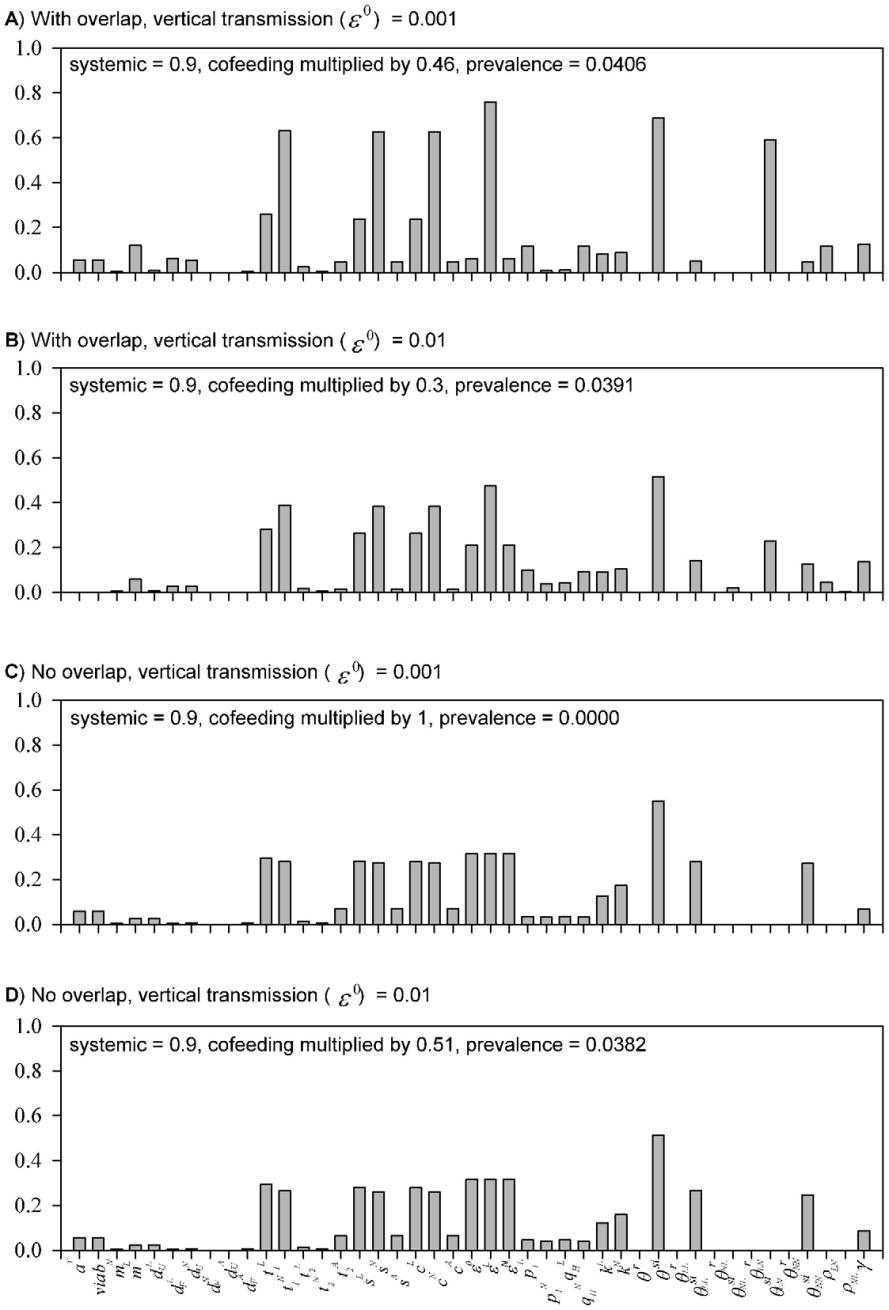
Relative importance of the input parameters. Elasticity values of the input parameters with inter-cohort overlap (A, B) and without overlap (C, D). Case 1 – lower vertical and higher cofeeding transmission – the parameters related to nymphs have much higher elasticities, indicating that nymphs play the critical role in determining R_0_. The cofeeing transmission parameter (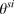) has high elasticity, and it is dominated by the pathway from nymphs to larvae (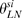). Case 2 – higher vertical and lower cofeeding transmission – the parameters for larvae increase elasticities.

For Case 1 with overlap, the elasticities of the parameters related to nymphs (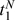, 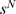, 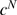, 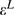) are much higher, indicating that nymphs play the critical role in determining R_0_ (Fig. 4A). The cofeeding transmission parameter (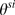) has high elasticity, and it is dominated by the pathway from nymphs to larvae (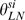). For Case 2 with overlap, the elasticities of the parameters for larvae (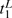, 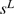, 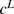, 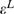) increase at the expense of those for nymphs (Fig. 4B). Overall, some of the relative importance shifts from cofeeding transmission to vertical (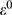) and trans-stadial transmission (pathogens are maintained through the moulting process) from nymphs to adults (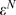). Furthermore, the elasticity of 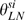 decreases, and instead contributions from other cofeeding transmission routes become more or less equal (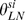, 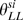, 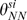). For the cases with no overlap, the contributions from larvae and nymphs further equalize. While cofeeding transmission remains as important, inter-cohort transmission has no effects due to the removal of cohort overlap (Fig. 4C, D).

## Discussion

Tick-borne flaviviruses are known to occur at relatively consistent, low prevalence in tick populations [11], [12]. In northern Wisconsin, the prevalence of POWV is usually between 1 and 5% [10], [12]. The mechanisms that lead to stable perpetuation of tick-borne flaviviruses in nature have not been fully described and are poorly understood because measuring various transmission rates under field conditions is technically and logistically challenging. Therefore, dynamic models of relevant transmission systems are useful exploratory and predictive tools. We incorporated tick seasonality into a comprehensive epidemiological model to examine the relative importance of all proposed transmission routes and the effects of overlapping tick cohorts on pathogen persistence. Our results show that cofeeding transmission is critically important for POWV to persist over a reasonable range of vertical transmission rates (Fig. 2). In the north-eastern and north central United States, where *I. scapularis* larvae and nymphs can overlap, resulting in some individuals from each stage feeding on the same animal [34], cofeeding transmission alone can be a sufficient mechanism to maintain POWV at observed prevalences.

**Figure 5:**
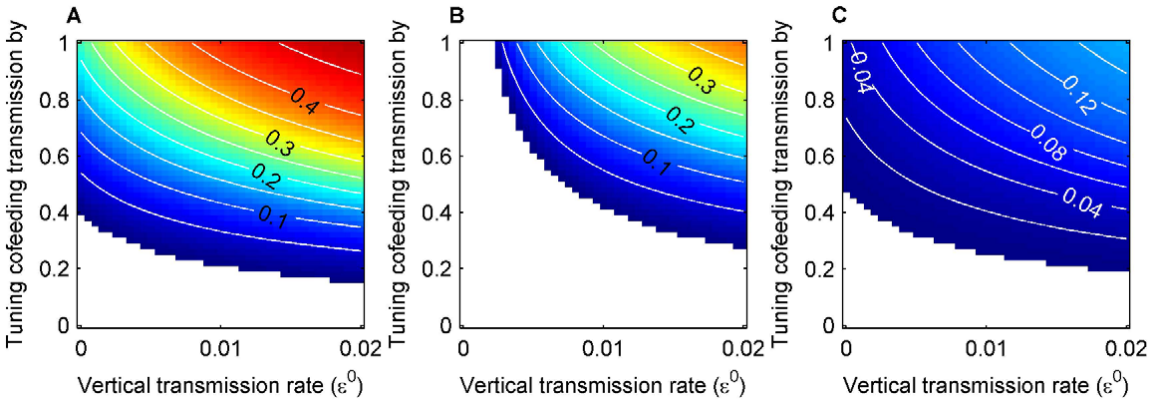
Prevalence of the pathogen with and without inter-cohort overlap for adults (A, B) and nymphs (C). 5A and 5B show the prevalence in adult ticks in October with inter-cohort overlap and without overlap, respectively. 5C shows the prevalence in nymphs in July with overlap. Systemic transmission is set at 0.9. These figures suggest that low, steady prevalence (>0–5%) in tick-borne flaviviruses could be maintained by low vertical, intermediate cofeeing, and high systemic transmission rates.

For a model to reasonably represent a robust tick-pathogen system, it should predict a sufficiently large region of the observed prevalence in the plausible parameter space. As we increased the systemic transmission rate to the observed level of 90% [41], the region of >0–5% prevalence becomes larger and occurs at intermediate cofeeding transmission rates (Fig. 5A, B). This suggests that the cofeeding transmission rate in the field must be moderately stable at intermediate levels and highlights that the cofeeding transmission rate is likely considerably lower than the measured rates from the laboratory experiments reported in [14]. Therefore, the model predicts that low, steady prevalence in tick-borne flaviviruses could be maintained by intermediate cofeeding and high systemic transmission rates (Fig. 5A). The result indicates that prevalence is relatively less sensitive to variation in the vertical transmission rate as long as the cofeeding transmission rate is stable at intermediate levels (Fig. 5A). However, when the cofeeding transmission rate is high, an increase in the vertical transmission rate could elevate prevalence substantially. When tick cohorts do not overlap (i.e. larvae and nymphs do not cofeed), this region of the parameter space can lead to zero prevalence (Fig. 5B). Hence, this set of parameter values is also consistent with the explanation that intermittent patterns of TBEV occurrences are due to the “fragile” link between cofeeding larvae and nymphs [30], [42], [43], which could be a result of either no overlap between cohorts, or larvae and nymphs feeding on different hosts. Vertical transmission becomes a crucial inter-generation link for pathogen persistence when larvae and nymphs do not overlap.

Nymphs of *I. scapularis* are responsible for most human infections with tick-borne diseases in North America because they frequently attack humans, are small and difficult to detect and may be extremely abundant in infested sites. However, observational data on the prevalence of POWV in nymphs does not exist. With the same parameter sets as in Fig. 5A, the model predicts that prevalence of ∼5% in adults corresponds to <∼1% prevalence in nymphs throughout their active season (Fig. 5C). Only a very small fraction of larvae are infected, well below 0.1% (results not shown). Although the lower proportion of infected larval ticks ultimately corresponds to a larger absolute number of infected individuals, nymphal ticks are probably more likely to transmit POWV to humans due to more frequent contact. Indeed, the month of onset of most recent POWV cases corresponds to peak periods of nymphal *I. scapularis* activity [4]. Our results suggest that efforts to prevent human POWV infection, like those for other tick-borne infections, should focus on reducing contact between *I. scapularis* nymphs and humans.

The major aim of this paper was to understand how tick seasonality influences the relative importance of pathogen transmission routes. By focusing on tick seasonality, the main limitation of our model is that we assumed an average, constant host population density. It is known that rodent populations, in particular, fluctuate both inter- and intra-annually [44]. Host density affects tick population size, and high host density can lead to dilution effects [45], [29]. In our model, dilution effects can be caused by either high density of incompetent hosts or of competent hosts due to reduced cofeeding transmission efficiency by less aggregation of ticks (Fig. S4; [29]). However, the effects of fluctuations in host density will largely depend on the relative timing of population peaks in ticks and hosts. Future studies should investigate how variations in host density might interact with tick seasonality to affect pathogen persistence.

Existing studies that examine models of tick-borne pathogens [24]–[29], [31] rarely report predicted prevalence. Ogden et al. [27] developed a model with tick seasonality and predicted prevalence of 6–9% in nymphal ticks for *Anaplasma phagocytophilum*, another pathogen with a relatively short infectivity period in the mouse host (14 days). Although their model does not include vertical transmission, the predicted prevalence is more or less comparable to ours (∼1%; Fig 5C) after roughly taking into account the difference in infectious period (14 vs. 3 days). However, if cofeeding transmission rates in the field are lower than the measured values from laboratory studies (and possibly than the values used in [27]), our model predicts that vertical transmission plays an important role in the persistence of pathogens with short infectivity in mammalian hosts. The work by Ogden et al. [27] and our results indicate the need for further studies.

**Figure 6:**
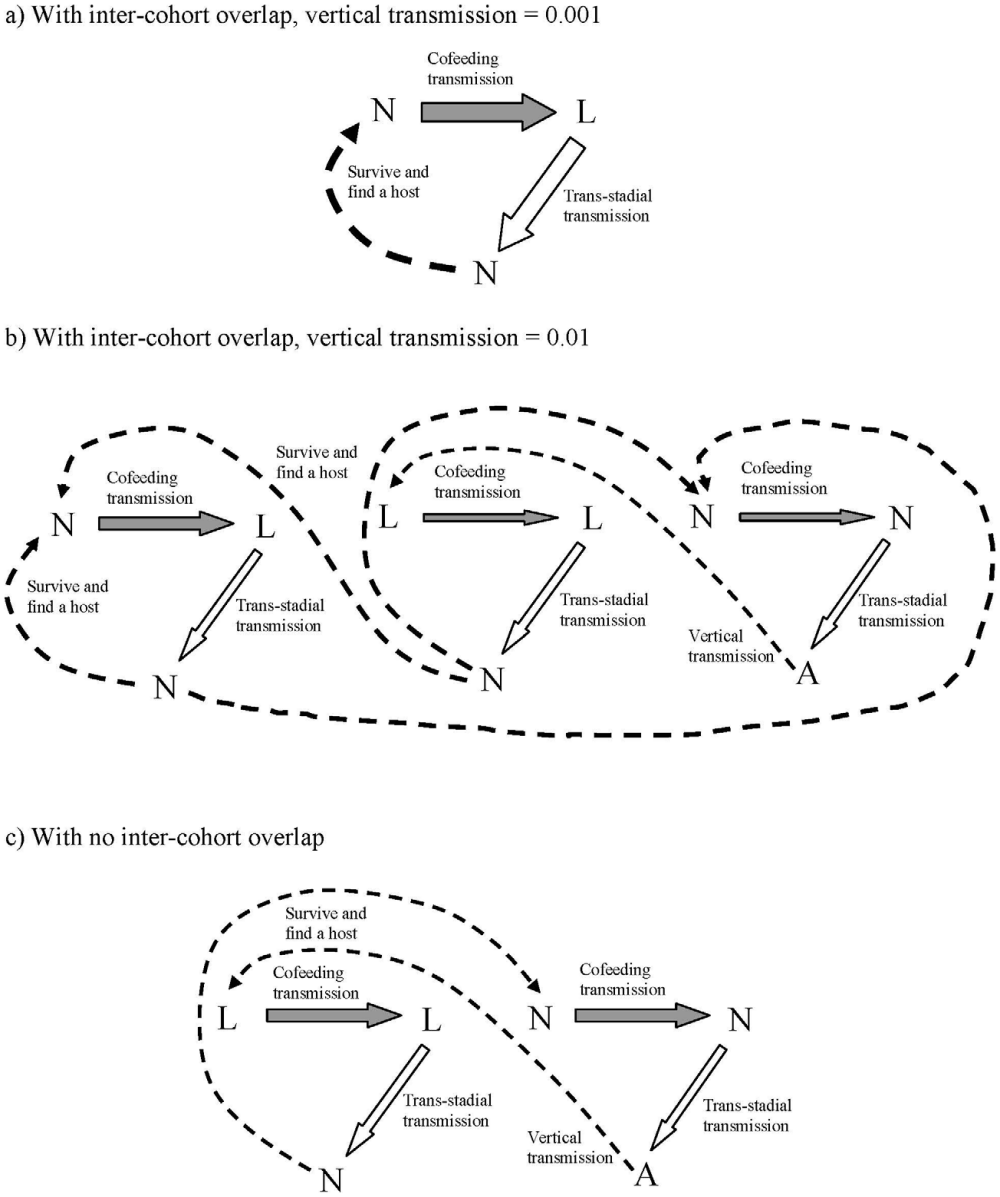
The proposed major transmission paths based on the elasticity analysis. The relative thickness of arrows qualitatively reflects the magnitudes of elasticity. When cohorts with the vertical transmission rate at 0.1% overlap (A), inter-cohort cofeeding transmission serves as the major route. With higher vertical transmission (1%), in addition to the above route, the importance of the two intra-cohort cofeeding transmission routes increases (B). When inter-cohort overlap is removed, the larva-nymph cofeeding route disappears, and the intra-cohort routes will maintain the pathogen with increased importance of the survival of the pathogen within ticks (C).

Although our model is motivated by the transmission of POWV in North America, elasticity analysis of the model parameters can give further insight into the mechanisms contributing to persistence of tick-borne flaviviruses in general. Both *I. scapularis and I. ricinus* are three-host ticks (ticks that feed on three hosts, one during each life stage), belong to the *I. ricinus* species complex, are closely related [46], and share similar ecology [47]. Although the model was parameterized mostly using values from *I. scapularis* studies, as far as we were able to compare, the parameter values are quite similar (e.g., compared to values used in [31]). Fig. 6 sketches the major mechanisms of persistence suggested by our model. When cohorts with the vertical transmission rate at 0.1% overlap, inter-cohort cofeeding transmission serves as the major route (Fig. 6A): the pathogen is transmitted from nymphs to larvae via cofeeding, the infected larvae become infected nymphs (via trans-stadial transmission), which then transmit the pathogen to the larvae of the next generation. In this scenario, nymphs become the integral part of the transmission process. With higher vertical transmission (1%), in addition to the above route, the importance of the two intra-cohort cofeeding transmission routes increases (Fig. 6B). Larvae and nymphs infected through these routes will feedback to other routes, reinforcing the entire cycle. Hence, when vertical transmission is higher, multiple feedback loops support pathogen persistence, which may make persistence more robust to external perturbations. When inter-cohort overlap is removed, the larva-nymph cofeeding route disappears, and the intra-cohort routes will maintain the pathogen with increased importance of the survival of the pathogen within ticks (Fig. 6C). This figure signifies that vertical transmission is crucial for pathogen persistence when the overlap is removed; once the link from adults to larvae via vertical transmission is removed, the entire pathogen maintenance cycle will collapse.

These results can shed some light on two opposing views regarding TBEV persistence in the European tick, *I. ricinus*, in the literature. One view is that vertical transmission is negligible and cofeeding transmission between larvae and nymphs is the key mechanism for TBEV persistence [17], [30]. The other view stresses the importance of vertical transmission and intra-cohort (i.e., larvae – larvae) cofeeding transmission and deemphasizes the effect of inter-cohort cofeeding transmission [22]. Vertical transmission and inter-cohort cofeeding are alternative trans-generational pathways of pathogen transmission. Our model indicates that which hypothesis is more plausible depends on the assumed value of vertical transmission and highlights the need for accurate measurements of vertical transmission rates under field conditions to evaluate the two propositions. One caveat is that the life history of *I. ricinus* can be more complex and could lead to more complex seasonal dynamics of the cohorts [20], [47]. Future modeling studies that investigate the significance of more complex seasonal cohort dynamics as well as more accurate measurements of transmission rates from field studies may be needed for understanding the relative importance of different transmission routes for pathogen persistence in the European tick populations.

**Figure 7:**
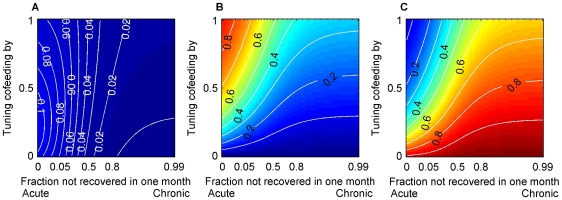
Relative importance of the three transmission routes with varied host recovery rates (γ). Relative importance of the three transmission routes with varied host recovery rates (γ); A) vertical, B) cofeeding, and C) systemic transmission. Cofeeding transmission rate was varied on the y-axis as described in the main text and specified in Table 1 and Table S1. Vertical transmission rate = 0.001 and systemic transmission rate = 0.9. Note that the scale of the x-axes is not linear. These figures suggest that the contributions from cofeeding transmission to pathogen persistence can be substantial even for pathogens with long infectious periods.

**Figure 8:**
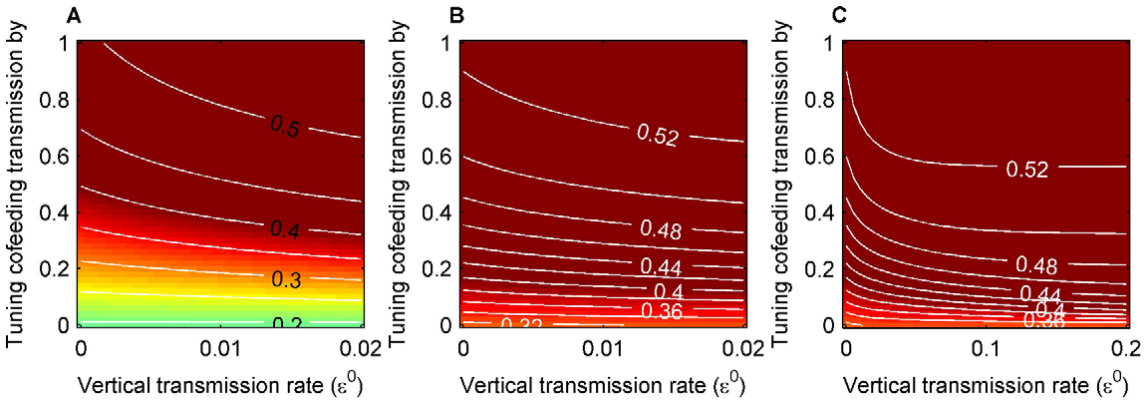
The effects of recovery rates on pathogen prevalence in adult ticks in October. Pathogen prevalence under longer infectious periods; A) about 4 months (γ = 0.03), B) chronic infection (γ = 0.001). Cofeeding and vertical transmission rates were varied, and systemic transmission was set at 0.9. Even when infection is chronic in hosts, cofeeding transmission can elevate prevalence. Recovery rates have larger effects when the cofeeding transmission rate is low. 8C shows prevalence of a pathogen that causes chronic infections in its host and has a relatively high vertical transmission rate, such as the agent of Lyme disease (the x-axis goes from 0 to 0.2 for this panel).

Finally, if we extrapolate our analysis to pathogens with longer periods of host infectivity, we find that the elasticity value for host recovery rate (γ) is not as important as we expected, at least close to the set of parameter values used. We initially thought that cofeeding transmission would not significantly contribute when host infectious periods are long, because pathogens would have plenty of opportunities to be transmitted via systemic transmission [48]. On the contrary, more detailed examination reveals that the contributions from cofeeding transmission to pathogen persistence are substantial even for infections with very long periods of infectiousness (Fig. 7). Specifically, the model predicts that cofeeding transmission can substantially increase pathogen prevalence even when host recovery rate is set at a small value, although cofeeding transmission is no longer necessary for pathogen persistence (Fig. 8A, B). To apply this to Lyme disease, which causes chronic infections in rodent hosts, we computed prevalence with a much higher value of vertical transmission, as it is apparently the only considerably different parameter value between Lyme disease and TBEV cases in the simulations in [31]. The results show that cofeeding transmission can still increase prevalence (Fig. 8C). Reported prevalence for Lyme disease is typically around 0.4 (e.g., [10]), suggesting that cofeeding transmission could be operating also in Lyme disease transmission cycles. Obviously, concrete conclusions require more rigorous examination and careful parameterization. At least, our model results question the common assumption that cofeeding transmission is unimportant in tick-borne pathogens causing long to chronic infectious periods [48]–[50]. In particular, many existing models of Lyme disease do not include cofeeding transmission and could underestimate pathogen prevalence [51].

## Supporting Information

Figure S1 Temporal dynamics of tick and host populations. Temporal dynamics of tick and host populations from a representative simulation run from the model. The number of infected host (H_1_) was multiplied by 10 prior to log_10_-transformation.
(0.98 MB TIF)

Figure S2 R_0_ of the pathogen over ranges of parameter values. R_0_ of the pathogen. See the figure legend for figure 2 in the article. When R_0_<1, the pathogen would not persist in the population following an initial invasion into a purely susceptible population of ticks and the hosts. R_0_ values well correspond to the prevalence levels.
(1.89 MB TIF)

Figure S3 Elasticity (relative importance) values when two cohorts do not overlap. Elasticity (relative importance) values when two cohorts do not overlap for vertical (left), (intra-cohort) cofeeding (middle), and systemic transmission (right) with the vertical transmission rate = 0.01, with (top) and without inter-cohort overlap (bottom). The pattern is similar to the cases with vertical transmission rate = 0.001 (Fig. 3 in the article).
(2.02 MB TIF)

Figure S4 Prevalence in adult ticks in October as the densities of the competent (H_1_) and incompetent (H_2_) hosts change. Pathogen prevalence varies over the densities of the two host species. The reduced prevalence in the lower right corner and upper left indicate the two types of dilution effects. The host density combination used in the model simulations is indicated by a pentagram.
(0.61 MB TIF)

Table S1 Table 1 in the main text with data sources. We augmented Table 1 in the main text with the citations of data sources for each parameter.
(0.17 MB DOC)

Table S2 The next generation matrix for the case with cohort overlap. The figure shows the blocks of elements which represent one of the transmission pathways. If the block contains a zero, all the elements in the block are zero.
(0.05 MB DOC)

Supporting Information S1 Descriptions and equations. In this document, we describe the model in more detail and provide the equations used in the computation.
(0.38 MB DOC)
