# Supplementary figures and images for: Persistence of Pathogens with Short Infectious Periods in Seasonal Tick Populations: The Relative Importance of Three Transmission Routes
Source: PLoS One. 2010 Jul 23;5(7):e11745. doi: 10.1371/journal.pone.0011745 (PMC2909195; doi:10.1371/journal.pone.0011745)

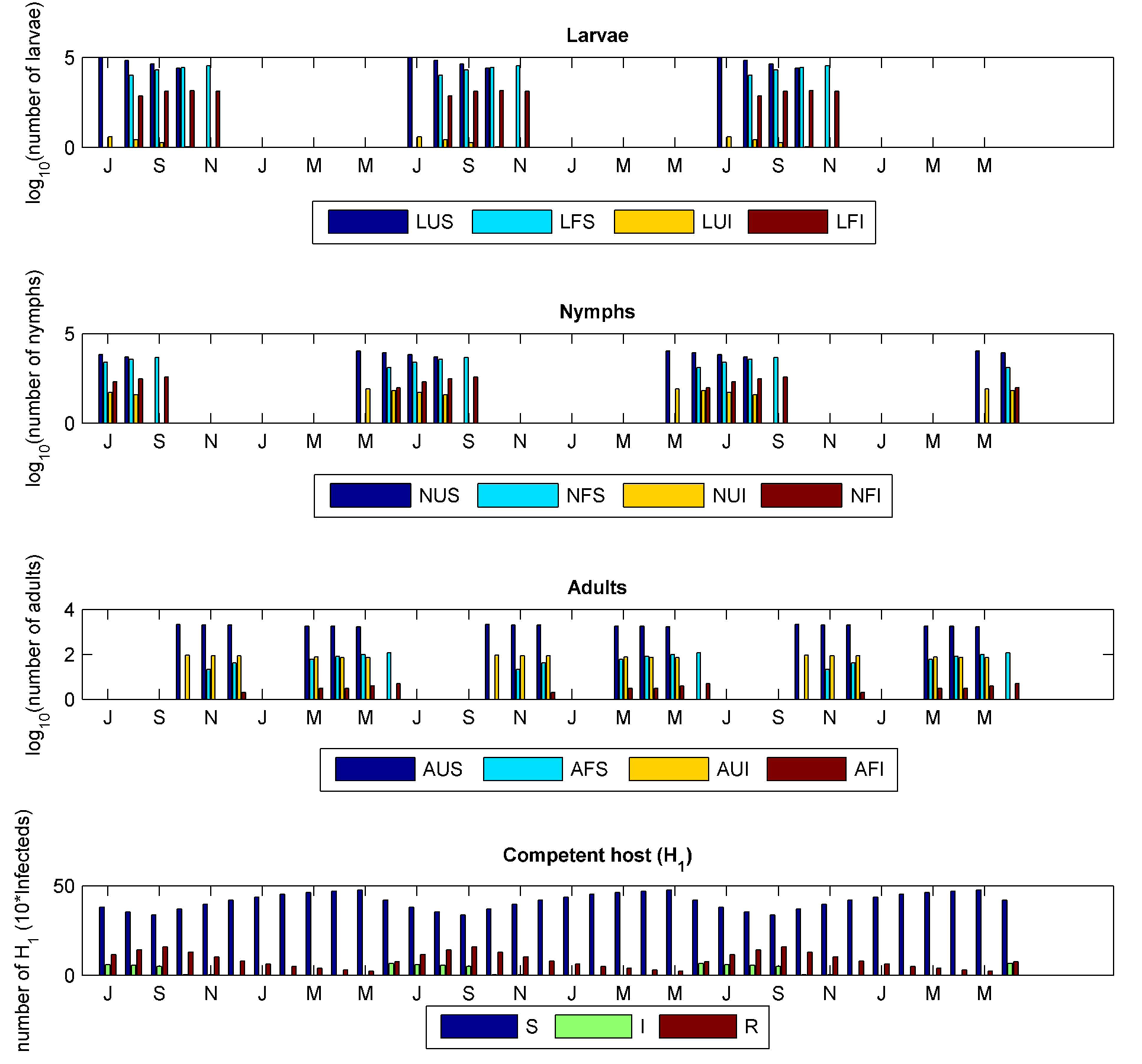

Supplement: Figure S1 — Temporal dynamics of tick and host populations. Temporal dynamics of tick and host populations from a representative simulation run from the model. The number of infected host (H1) was multiplied by 10 prior to log10-transformation. (0.98 MB TIF) [file pone.0011745.s001.tif]

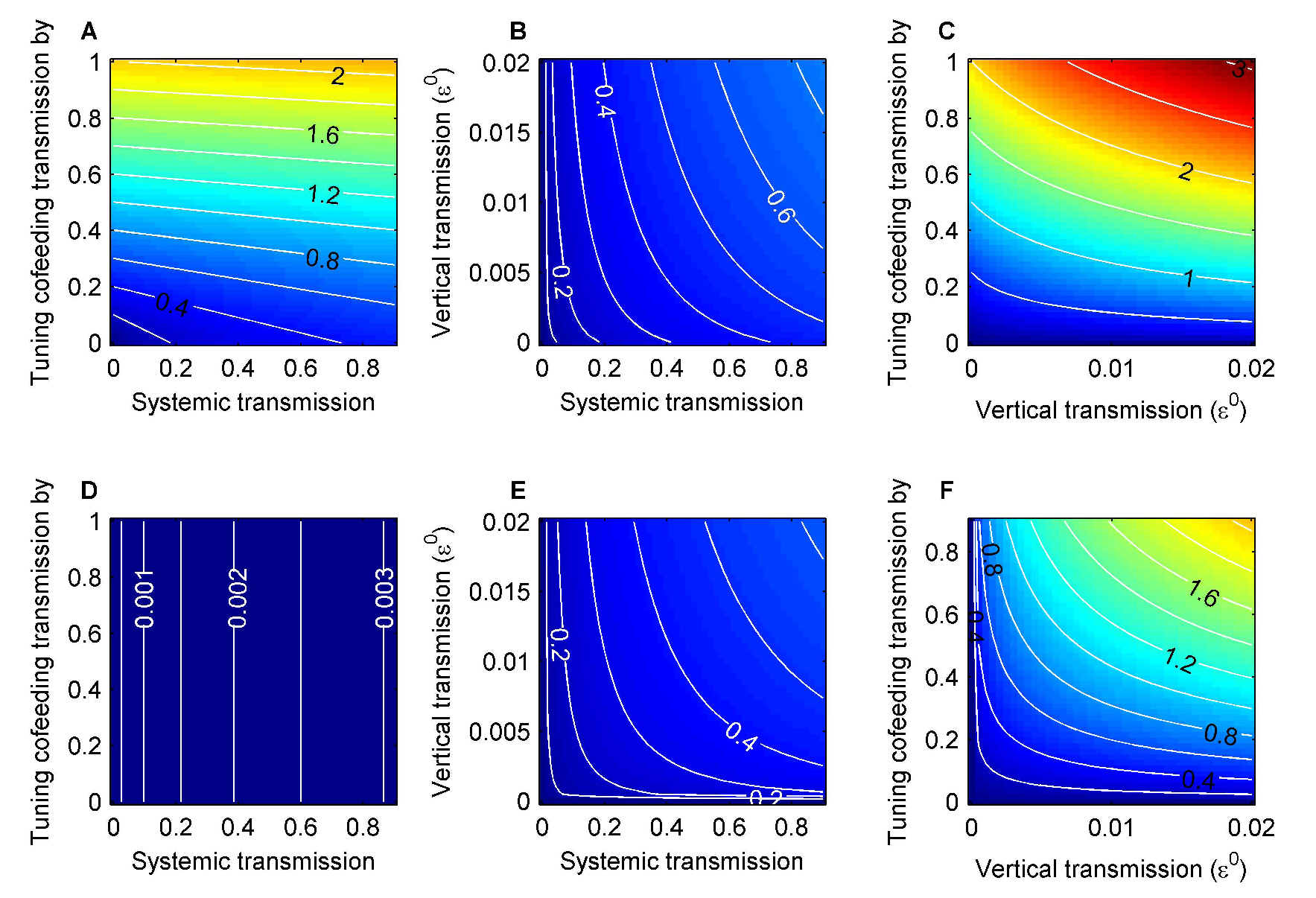

Supplement: Figure S2 — R0 of the pathogen over ranges of parameter values. R0 of the pathogen. See the figure legend for figure 2 in the article. When R0<1, the pathogen would not persist in the population following an initial invasion into a purely susceptible population of ticks and the hosts. R0 values well correspond to the prevalence levels. (1.89 MB TIF) [file pone.0011745.s002.tif]

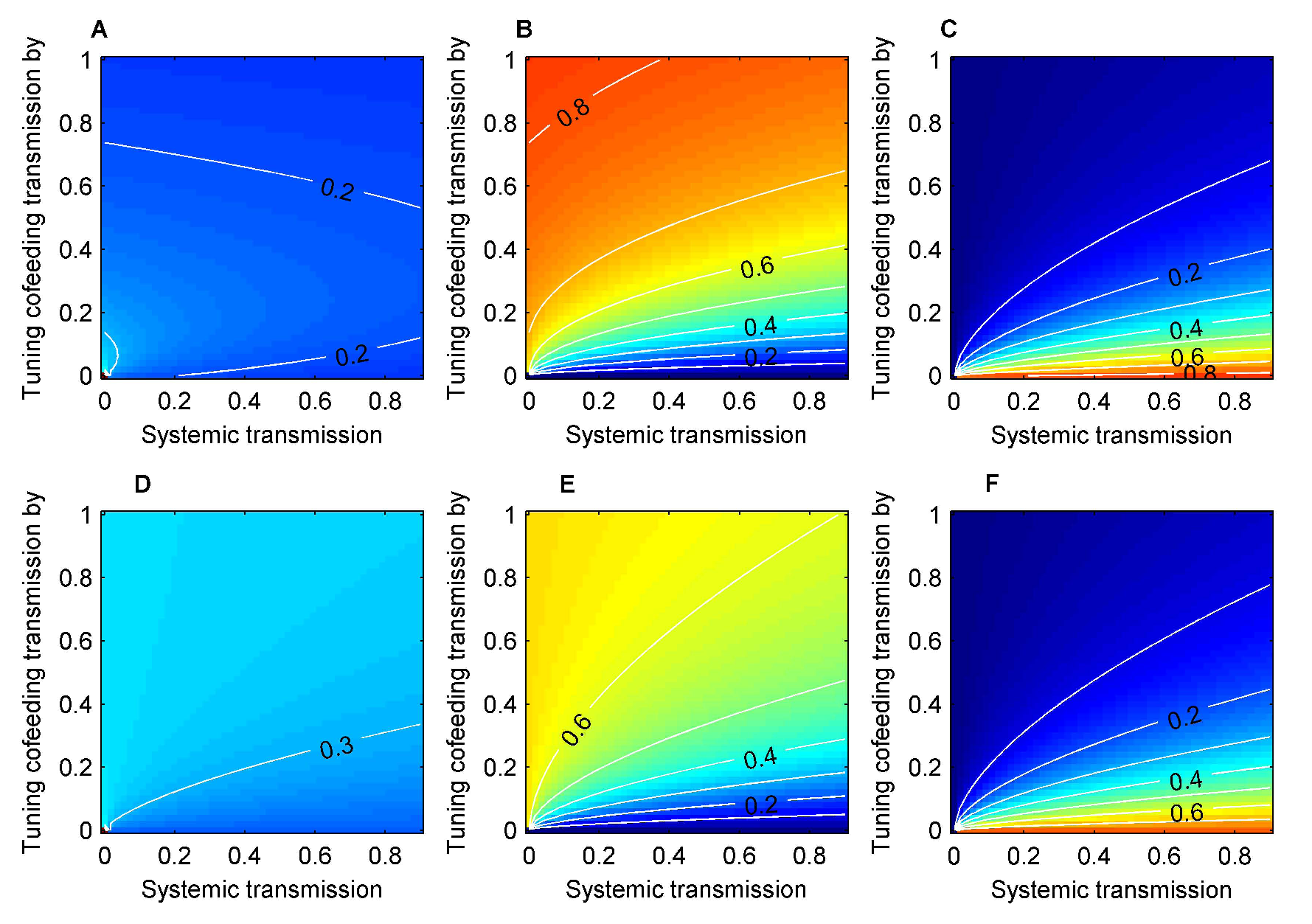

Supplement: Figure S3 — Elasticity (relative importance) values when two cohorts do not overlap. Elasticity (relative importance) values when two cohorts do not overlap for vertical (left), (intra-cohort) cofeeding (middle), and systemic transmission (right) with the vertical transmission rate = 0.01, with (top) and without inter-cohort overlap (bottom). The pattern is similar to the cases with vertical transmission rate = 0.001 (Fig. 3 in the article). (2.02 MB TIF) [file pone.0011745.s003.tif]

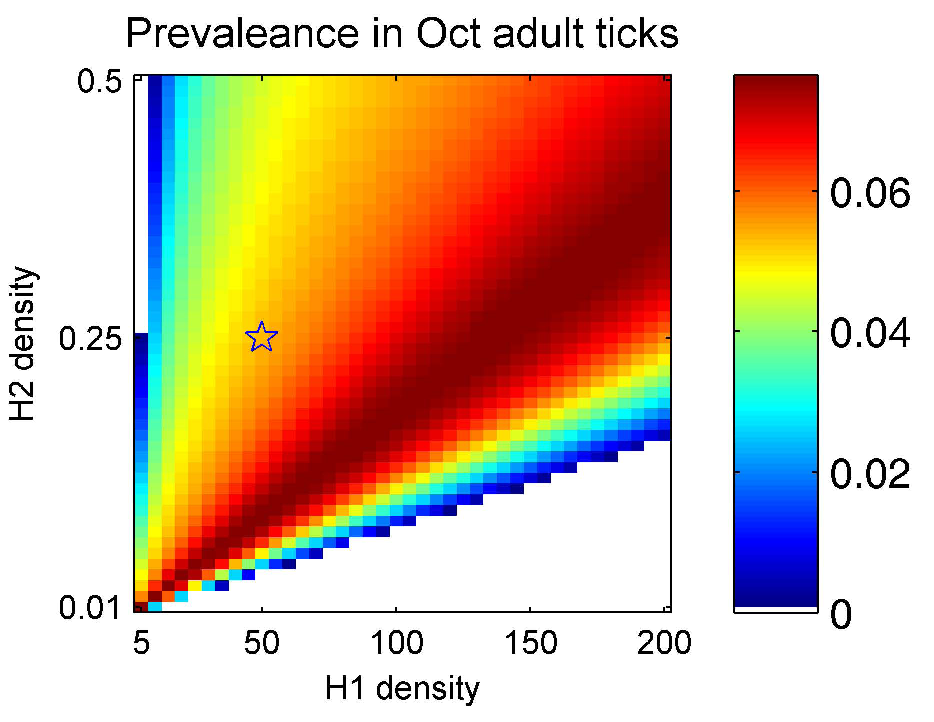

Supplement: Figure S4 — Prevalence in adult ticks in October as the densities of the competent (H1) and incompetent (H2) hosts change. Pathogen prevalence varies over the densities of the two host species. The reduced prevalence in the lower right corner and upper left indicate the two types of dilution effects. The host density combination used in the model simulations is indicated by a pentagram. (0.61 MB TIF) [file pone.0011745.s004.tif]
