# Supplementary material for: Persistence of Pathogens with Short Infectious Periods in Seasonal Tick Populations: The Relative Importance of Three Transmission Routes
Source: PLoS One. 2010 Jul 23;5(7):e11745. doi: 10.1371/journal.pone.0011745 (PMC2909195; doi:10.1371/journal.pone.0011745)
Supplement: Table S1 — Table 1 in the main text with data sources. We augmented Table 1 in the main text with the citations of data sources for each parameter. (0.17 MB DOC) [file pone.0011745.s005.doc]

**Appendix S5. Parameter definitions, the values used in the simulations and the literature sources.** All the rates are per month. Parameters with an asterisk (*) were varied in simulations (the ranges in brackets). The last column was added to Table 1 in the article.

| Parameter | Definition | | Value | Source | | Species |
| --- | --- | --- | --- | --- | --- | --- |
| $a_{T}$ | Number of eggs laid per female tick | | 2000 | 1,2 | | I. scapularis, I. ricinus |
| $viab$ | Fraction of eggs that produce a viable, active larva | | 0.4 | 2,3,4 | | I. scapularis, I. ricinus |
| $m^{N}$ | Probability of female nymph-to-adult moulting | | 0.5∙exp(-0.09) | 50/50 sex ratio; Assume mostly die from starvation; 1, 3 | | I. scapularis |
| $m^{L}$ | Probability of larva-to-nymph moulting | | exp(-0.9) | 5 | | I. scapularis |
| $d_{U}^{L}$ | Mortality rate of unfed larvae | |  | | 4,6 | I. scapularis, I. ricinus* |
| $d_{F}^{L}$ | Mortality rate of fed larvae | |  | | 4,6 | I. scapularis, I. ricinus* |
| $d_{U}^{N}$ | Mortality rate of unfed nymphs | |  | | 4,6 | I. scapularis, I. ricinus* |
| $d_{F}^{N}$ | Mortality rate of fed nymphs | |  | | 4,6 | I. scapularis, I. ricinus* |
| $d_{U}^{A}$ | Mortality rate of unfed adults | |  | | 4,6 | I. scapularis, I. ricinus* |
| $d_{F}^{A}$ | Mortality rate of fed adults | |  | | 4,6 | I. scapularis, I. ricinus* |
| $d_{H}$ | Mortality rate of host 1 (H_1_) | | exp(-0.25) | Longevity of mice is about 1 year; 4 | |  |
| $t_{1}^{L}$ | Number of larvae on a H_1_ individual | | 27.8 | 5 | | I. scapularis |
| $t_{1}^{N}$ | Number of nymphs on a H_1_ individual | | 5 | 7-9 | | I. scapularis |
| $t_{1}^{A}$ | Number of adults on a H_1_ individual | | 0 | By assumption | |  |
| $t_{2}^{L}$ | Number of larvae on a H_2_ individual | | 239 | 5 | | I. scapularis |
| $t_{2}^{N}$ | Number of nymphs on a H_2_ individual | | 20 | 1,10,11,12 | | I. scapularis |
| $t_{2}^{A}$ | Number of adults on a H_2_ individual | | 30 | ,1,12,15 | | I. scapularis |
| $s^{L}$ | Number of questing days per month of larvae | | 30 | Based on 1 | | I. scapularis |
| $s^{N}$ | Number of questing days per month of nymphs | | 30 | Based on 1 | | I. scapularis |
| $s^{A}$ | Number of questing days per month of adults | | 30 | Based on 1 | | I. scapularis |
| $c^{L}$ | Number of days larvae remain attached on a host | | 3 | 1,16,17 | | I. scapularis, I. ricinus |
| $c^{N}$ | Number of days nymphs remain attached on a host | | 5 | 1,,16,17 | | I. scapularis, I. ricinus |
| $c^{A}$ | Number of days adults remain attached on a host | | 10 | 1,16 | | I. scapularis, I. ricinus |
| $\gamma$ | Recovery rate of H_1_ | | 0.3∙(30) | 21, By assumption that hosts are viremic only for 2 – 3 days | |  |
| $\varepsilon^{0}$ | Probability of vertical transmission | | 0.001* | 18,19 | | I. ricinus |
| $\varepsilon^{L}$ | Probability of larva-nymph trans-stadial transmission | | 0.22 | 20 | | I. scapularis |
| $\varepsilon^{N}$ | Probability of nymph-adult trans-stadial transmission | | 0.54 | 18 | | I. scapularis |
| $p_{1}^{L}$ | Probability of H_1_-to-larva systemic transmission | | 0.9*, 0.8  [0, 0.9] | 20,21 | | I. scapularis, I. ricinus |
| $p_{1}^{N}$ | Probability of H_1_-to-nymph systemic transmission | | 0.9*, 0.8  [0, 0.9] | 20,21 | | I. scapularis, I. ricinus |
| $q_{H}^{L}$ | Probability of larva-to-H_1_ systemic transmission | | 0.8, 0.9 | 20,21 | | I. scapularis, I. ricinus |
| $q_{H}^{N}$ | Probability of nymph-to-H_1_ systemic transmission | | 0.8, 0.9 | 20,21 | | I. scapularis, I. ricinus |
| $q_{H}^{A}$ | Probability of adult-to-H_1_ systemic transmission | | 0 | By assumption;20,21 | | I. scapularis, I. ricinus |
| $k^{L}$ | Aggregation parameter of the negative binomial distribution for larvae | | 1.19 | 22 | | I. scapularis |
| $k^{N}$ | Aggregation parameter of the negative binomial distribution for nymph | | 0.56 | 22 | | I. scapularis |
| $\rho_{LL}$ | Correlation coefficient of larvae | | 1 | By the model assumption; 23,24 | |  |
| $\rho_{LN}$ | Correlation coefficient of larvae and nymphs | | 0.2 | 22 | | I. scapularis |
| $\rho_{NN}$ | Correlation coefficient of nymphs | | 1 | By the model assumption; 23,24 | |  |
| $\theta^{r}$ $\left(\theta_{LL}^{r},\theta_{LN}^{r},\theta_{NL}^{r},\theta_{NN}^{r}\right)$ | | Probability of cofeeding transmission on a recovered H_1_ | 0.24*  [0, 0.24] | 25; Assumed cofeeding transmission the same between any stages of ticks | | I. ricinus |
| $\theta^{si}$ $\left(\theta_{LL}^{si},\theta_{LN}^{si},\theta_{NL}^{si},\theta_{NN}^{si}\right)$ | | Probability of cofeeding transmission on a susceptible or infected H_1_ | 0.72*  [0, 0.72] | 25; Assumed cofeeding transmission the same between any stages of ticks | | I. ricinus |

* We used mortality rates for *I. ricinus* because the sources for mortality rates for *I. scapularis* used in [4] were unclear. The mortality rates reported in [2] and [4] are somewhat different (maximum of one order of magnitude). We believe that the differences would not change our results significantly since mortality rates turned out to be minor according to the elasticity analyses and most of ticks die from starvation.

**References**

1. Sandberg S, Awebuch TE, Spielman A. (1992) A comprehensive multiple matrix model representing the life cycle of the tick that transmits the agent of Lyme disease. Journal of Theoretical Biology 157: 203-220.
2. Randolph SE. (2004) Tick ecology: processes and patterns behind the epidemiological risk posed by ixodid ticks as vectors. Parasitology 129: S37–S65.
3. Lindsay RL, Barker IK, Surgeoner GA, McEwen SA, Gillespie TJ, Addison EM. (1998) Survival and development of the different life stages of *Ixodes scapularis* (Acari: Ixodidae) held within four habitats on Long Point, Ontario, Canada. Journal of Medical Entomology 35: 189-199.
4. Ogden NH, Lindsay LR, Beauchamp G, Charron D, Maarouf A, O’Callaghan CJ, Waltner-Toews D, Barker IK. (2004) Investigation of relationships between temperature and developmental rates of tick *Ixodes scapularis* (Acari: Ixodidae) in the laboratory and field. Journal of Medical Entomology 41: 622-633.
5. LoGiudice K, Ostfeld RS, Schmidt KA, Keesing F. (2003) The ecology of infectious disease: Effects of host diversity and community composition on Lyme disease risk. Proceedings of the National Academy of Sciences of America (PNAS) 100: 567–571.
6. Lackey JA, Huckaby DG, Ormiston BG. (1985) *Peromyscus leucopus*. In Mammalian species, vol. 247, pp. 1-10.
7. Ostfeld RS, Miller MC, Hazler KR. (1996) Causes and consequences of tick (*Ixodes scapularis)* burdens on white-footed mice (*Peromyscus leucopus*). Journal of Mammalogy 77: 266-273.
8. Proco TC. (1999) A mathematical model of the ecology of Lyme disease. IMA Journal of Mathematics Applied in Medicine and Biology 16: 261-296.
9. Schmidt K, Ostfeld R, Schauber E. (1999) Infestation of *Peromyscus leucopus* and *Tamias striatus* by *Ixodes scapularis* (Acari : Ixodidae) in relation to the abundance of hosts and parasites. Journal of Medical Entomology 36: 749-757.
10. Piesman J, Spielman A. (1979) Host associations and seasonal abundance of immature Ixodes dammini in southeastern Massachusetts, USA. Annals of the Entomological Society of America 72: 829-832.
11. Telford SR III, Mather TN, Moore SI., Wilson ML, Spielman A. (1988) Incompetence of deer as reservoirs of the Lyme disease spirochete. Am. J. Trop. Med. Hyg. 39: 105-109.
12. Madhav NK, Brownstein JS, Tsao J, Fish D. (2004) A dispersal model for the range expansion of blacklegged tick (Acari: Ixodidae). Journal of Medical Entomology 41: 842–852.
13. Anderson JF, Magnarelli LA. (1980) Vertebrate host relationships and distribution of ixodid ticks (Acari: Ixodidae) in Connecticut, USA. Journal of Medical Entomology 17: 314-323.
14. Wilson ML, Litwin TS, Gavin TA, Capkanis MC, MacLean DC, Spielman A. (1990) Host-dependent differences in feeding and reproduction of *Ixodes dammini* (Acari: Ixodidae). Journal of Medical Entomology 27: 945-954.
15. Schulze TL, Jordan RA, Hung RW, Schulze CJ. (2009) Effectiveness of the 4-poster passive tropical treatment device in the control of *Ixodes scapularis* and *Amblyomma americanum* (Acari: Ixodidae) in New Jersey. Vector-borne and Zoonotic Diseases 9, 389-400.
16. Mather TN, Spielman A. (1986) Diurnal detachment of immature deer ticks (*Ixodes dammini*) from nocturnal hosts. Am J. Trop. Med. Hyg. 35: 182-186.
17. Randolph SE, Craine NG. (1995) General framework for comparative quantitative studies on transmission of tick-borne diseases using Lyme borreliosis in Europe as an example. Journal of Medical Entomology 32: 765-777.
18. Costero A, Grayson MA. (1996) Experimental trasmission of powassan virus (Flaviviridae) by *Ixodes scapularis* ticks (Acari: Ixodidae). Am. J. Trop. Med. Hyg. 55: 536-546.
19. Danielova V, Holubova H, Pejcoch M, Daniel M. (2002) Potential significance of transovarial transmission in the circulation of tick-borne encephalitis. Folia Parasitologica 49: 323-325.
20. Ebel GD, Kramer LD. (2004) Short report: duration of tick attachment required for transmission of powassan virus by deer ticks. Am. J. Trop. Med. Hyg. 71: 268-271.
21. Hartemink NA, Randolph SE, Davis SA, Heesterbeek JAP. (2008) The basic reproduction number for complex disease systems: defining R_0_ for tick-borne infections. American Naturalist 171: 743–754.
22. Brunner JL, Ostfeld RS (2008) Multiple causes of variable tick burdens on small-mammal hosts. Ecology 89: 2259-2272.
23. Rosa R, Pugliese A, Norman R, Hudson PJ. (2003) Thresholds for disease persistence in models for tick-borne infections including non-viraemic transmission, extended feeding and tick aggregation. Journal of Theoretical Biology 224: 359-376.
24. Rosa R, Pugliese A. (2007) Effects of tick population dynamics and host densities on the persistence of tick-borne infections. Mathematical BioSciences 208: 216–240.
25. Labuda M, Kozuch O, Zuffova E, Eleckova E, Hails R et al. (1997) Tick-borne encephalitis virus transmission between ticks cofeeding on specific immune natural rodent hosts. Virology 235: 138-143.
