# Supplementary material for: Persistence of Pathogens with Short Infectious Periods in Seasonal Tick Populations: The Relative Importance of Three Transmission Routes
Source: PLoS One. 2010 Jul 23;5(7):e11745. doi: 10.1371/journal.pone.0011745 (PMC2909195; doi:10.1371/journal.pone.0011745)
Supplement: Table S2 — The next generation matrix for the case with cohort overlap. The figure shows the blocks of elements which represent one of the transmission pathways. If the block contains a zero, all the elements in the block are zero. (0.05 MB DOC) [file pone.0011745.s006.doc]

**Appendix S6. The next generation matrix for the case with cohort overlap**. The figure shows the blocks of elements which represent one of the transmission pathways. If the block contains a zero, all the elements in the block are zero. Egg, L, N, and A refer to tick life stages, and H to the competent host (H_1_). The numbers refer to months when corresponding ticks or H_1_ are active (e.g., 8 = August).

|  | Egg | L8 | L9 | L10 | L11 | N6 | N7 | N8 | N9 | H6 | H7 | H8 | H9 | H10 | H11 |
| --- | --- | --- | --- | --- | --- | --- | --- | --- | --- | --- | --- | --- | --- | --- | --- |
| Egg | vertical | vertical | | | | vertical | | | | 0 | | | | | |
| L8 | cofeeding Egg → L | cofeeding L → L | | | | 0 | | | | systemic H → L | | | | | |
| L9 |  |  |  |  |  |  |  |  |  |  |  |  |  |  |  |
| L10 |  | 0 | | | |  |  |  |  |  |  |  |  |  |  |
| L11 |  |  |  |  |  |  |  |  |  |  |  |  |  |  |  |
| N6 | cofeeding Egg → N | cofeeding L → N | | | | 0 | | | | systemic H → N | | | | | |
| N7 |  |  |  |  |  |  |  |  |  |  |  |  |  |  |  |
| N8 |  |  |  |  |  |  |  |  |  |  |  |  |  |  |  |
| N9 |  |  |  |  |  |  |  |  |  |  |  |  |  |  |  |
| H6 | systemic Egg → H | systemic L → H | | | | 0 | | | | 0 | | | | | |
| H7 |  |  |  |  |  |  |  |  |  |  |  |  |  |  |  |
| H8 |  |  |  |  |  |  |  |  |  |  |  |  |  |  |  |
| H9 |  |  |  |  |  |  |  |  |  |  |  |  |  |  |  |
| H10 |  | 0 | | | |  |  |  |  |  |  |  |  |  |  |
| H11 |  |  |  |  |  |  |  |  |  |  |  |  |  |  |  |
