# Supplementary material for: Persistence of Pathogens with Short Infectious Periods in Seasonal Tick Populations: The Relative Importance of Three Transmission Routes
Source: PLoS One. 2010 Jul 23;5(7):e11745. doi: 10.1371/journal.pone.0011745 (PMC2909195; doi:10.1371/journal.pone.0011745)
Supplement: Supporting Information S1 — Descriptions and equations. In this document, we describe the model in more detail and provide the equations used in the computation. (0.38 MB DOC) [file pone.0011745.s007.doc]

**Appendix S4**

**The model**

The probability that a tick in life stage Z feeds is given by

$P_{f}^{Z}=1-\exp\left[-\left(\beta_{1}^{Z}H_{1}+\beta_{2}^{Z}H_{2}\right)\right]$, where $\beta_{h}^{Z}=\frac{t_{h}^{Z}\cdot s^{Z}}{Z_{U}\cdot c^{Z}}$, Z = L, N, A, h = 1, 2

(See Table 1 for parameter definitions). L, N, or A corresponds to the larva, nymph, or adult life stage of the tick, and 1 and 2 refer to the host species 1 (H_1_) and host species 2 (H_2_), respectively. $Z_{U}$is the number of unfed ticks in life stage Z. The probability that a tick is infected upon feeding on a host is modelled from simple probability rules:

$P_{\inf}^{Z}=P_{H1^{sr}}^{Z}\cdot P_{ct}^{Z}+P_{H1^{i}}^{Z}\cdot\left(P_{ct}^{Z}+P_{st}^{Z}\cdot\left(1-P_{ct}^{Z}\right)\right)$.

$P_{H1^{sr}}^{Z}$is the proportion of susceptible or recovered H_1_ hosts (i.e., non-viraemic), and $P_{H1^{i}}^{Z}$is the proportion of infected H_1_ hosts (i.e., viraemic) encountered by ticks of stage class Z. $P_{ct}^{Z}$and$P_{st}^{Z}$ are the probabilities of cofeeding and systemic transmission in ticks of stage class Z, respectively. Here we assume that cofeeding transmission is the only way in which ticks can be infected by feeding on susceptible or recovered hosts, and that cofeeding and systemic transmission are mutually exclusive but either is possible on infected hosts. For the cofeeding transmission rate, we adopt and modify the formulation used by Rosà and Pugliese [1] to include the immunity condition (i.e., recovered or not) of the competent host. We set the parameters such that cofeeding transmission on recovered hosts is more difficult than on susceptible or infected hosts as Labuda et al. [2] found in their experiments. The probability of cofeeding transmission to a larva from a larva becomes,

$$P_{LL}=1-\exp\left(-\frac{L_{Q,t}^{i}P_{f}^{L}\left(\lambda_{LL}^{r}P_{H1^{r}}^{L}+\lambda_{LL}^{si}P_{H1^{si}}^{L}\right)}{H_{1}}\right)$$

where $\lambda_{LL}^{r}=\theta_{LL}^{r}\left(1+\frac{\rho_{LL}}{\sqrt{k^{L}k^{L}}}\right)$ and $\lambda_{LL}^{si}=\theta_{LL}^{si}\left(1+\frac{\rho_{LL}}{\sqrt{k^{L}k^{L}}}\right)$

The λ’s are the probabilities of cofeeding transmission enhanced by aggregation of ticks (negative binomial). The parameter $k^{Z}$is the aggregation (or dispersion) parameter of the negative binomial distribution for ticks in life stage Z [3,4]. The parameter$\rho_{ZZ'}$ is the correlation between burdens of ticks in stage Z and those in stage Z’ on a host [5]. For example, when the same host individuals tend to harbour a large number of larvae and nymphs, the correlation $\rho_{LN}\left(=\rho_{NL}\right)$ is close to 1. If Z = Z’, correlation is identically 1. The probability of cofeeding transmission to a larva from a nymph is similarly,

$P_{LN}=1-\exp\left(-\frac{N_{Q,t}^{i}P_{f}^{N}\left(\lambda_{LN}^{r}P_{H1^{r}}^{N}+\lambda_{LN}^{si}P_{H1^{s}+H1^{i}}^{N}\right)}{H_{1}}\right)$.

Then the total probability of a larva to be infected via cofeeding transmission is

$P_{ct}^{L}=P_{LL}+P_{LN}\left(1-P_{LL}\right)$. The total probability of a nymph to be infected via cofeeding transmission is modeled similarly. For adult ticks, both the probabilities of systemic and cofeeding transmission are zero since they are assumed to feed only on the incompetent host which supports neither transmission route. The probability of not being infected upon feeding is the complement of that of being infected:

$$P_{non-\inf}^{Z}=1-P_{\inf}^{Z}=P_{H1^{sr}}^{Z}\cdot\left(1-P_{ct}^{Z}\right)+P_{H1^{i}}^{Z}\cdot\left(1-P_{st}^{Z}\right)\cdot\left(1-P_{ct}^{Z}\right)+P_{H2}^{Z}$$

The infection rate of the competent host is also modelled as mass action [1]:

$P{}_{\inf}^{H1}=1-\exp\left\{-\left(q_{H}^{L}\beta_{1}^{L}L_{Q,t}^{i}+q_{H}^{N}\beta_{1}^{N}N_{Q,t}^{i}+q_{H}^{A}\beta_{1}^{A}A_{Q,t}^{i}\right)\right\}$.

The host mortality rate was chosen to reflect longevity of about one year, which is typical in small rodents in the upper midwest to northeastern United States [6]. The birth rate was set so that the total host density remains constant.

**The model components and equations**

The rate of feeding on any host = $P_{f}^{Z}=1-\exp\left[-\left(\beta_{1}^{Z}H_{1}+\beta_{2}^{Z}H_{2}\right)\right]$

where $\beta_{h}^{Z}=\frac{t_{h}^{Z}\cdot que\_d^{Z}}{Z_{U}\cdot atch\_d^{Z}}$, Z = L, N, A, h = 1, 2

L, N, or A corresponds to the larva, nymph, or adult life stage of the tick, and 1 and 2 refers to the host species 1 (H_1_) and host species 2 (H_2_), respectively.

Let $P_{st}$, $P_{ct}$the probability of systemic transmission and cofeeding transmission, respectively;

The rate of feeding but not being infected (*FEED_NOT_INFECTED^Z^*)

= $P_{f}^{Z}\cdot\left(P_{H1^{sr}}^{Z}\cdot\left(1-P_{ct}^{Z}\right)+P_{H1^{i}}^{Z}\cdot\left(1-P_{st}^{Z}\right)\cdot\left(1-P_{ct}^{Z}\right)+P_{H2}^{Z}\right)$

The rate of feeding and being infected (*FEED_AND_INFECTED^Z^*)

= $P_{f}^{Z}\cdot\left(P_{H1^{sr}}^{Z}\cdot P_{ct}^{Z}+P_{H1^{i}}^{Z}\cdot\left(P_{ct}^{Z}+P_{st}^{Z}\cdot\left(1-P_{ct}^{Z}\right)\right)\right)$

The rate of cofeeding transmission from a larva to a larva

= $P_{LL}$=$\left[1-\exp\left(-\frac{L_{Q,t}^{i}P_{f}^{L}\left(\lambda_{LL}^{r}P_{H1^{r}}^{L}+\lambda_{LL}^{si}P_{H1^{s}+H1^{i}}^{L}\right)}{H_{1}}\right)\right]$

The rate of cofeeding transmission from a nymph to a larva

= $P_{LN}$=$\left[1-\exp\left(-\frac{N_{Q,t}^{i}P_{f}^{N}\left(\lambda_{LN}^{r}P_{H1^{r}}^{N}+\lambda_{LN}^{si}P_{H1^{s}+H1^{i}}^{N}\right)}{H_{1}}\right)\right]$

The rate of cofeeding transmission from a nymph to a nymph

= $P_{NN}$=$\left[1-\exp\left(-\frac{N_{Q,t}^{i}P_{f}^{N}\left(\lambda_{NN}^{r}P_{H1^{r}}^{N}+\lambda_{NN}^{si}P_{H1^{s}+H1^{i}}^{N}\right)}{H_{1}}\right)\right]$

The rate of cofeeding transmission from a larva to a nymph

= $P_{NL}$=$\left[1-\exp\left(-\frac{L_{Q,t}^{i}P_{f}^{L}\left(\lambda_{NL}^{r}P_{H1^{r}}^{L}+\lambda_{NL}^{si}P_{H1^{s}+H1^{i}}^{L}\right)}{H_{1}}\right)\right]$

where,

$\lambda_{LL}^{r}=\theta_{LL}^{r}\left(1+\frac{\rho_{LL}}{\sqrt{k^{L}k^{L}}}\right)$ , $\lambda_{LL}^{si}=\theta_{LL}^{si}\left(1+\frac{\rho_{LL}}{\sqrt{k^{L}k^{L}}}\right)$

$\lambda_{LN}^{r}=\theta_{LN}^{r}\left(1+\frac{\rho_{LN}}{\sqrt{k^{L}k^{N}}}\right)$, $\lambda_{LN}^{si}=\theta_{LN}^{si}\left(1+\frac{\rho_{LN}}{\sqrt{k^{L}k^{N}}}\right)$

$\lambda_{NN}^{r}=\theta_{NN}^{r}\left(1+\frac{\rho_{NN}}{\sqrt{k^{N}k^{N}}}\right)$, $\lambda_{NN}^{si}=\theta_{NN}^{si}\left(1+\frac{\rho_{NN}}{\sqrt{k^{N}k^{N}}}\right)$

$\lambda_{NL}^{r}=\theta_{NL}^{r}\left(1+\frac{\rho_{NL}}{\sqrt{k^{N}k^{L}}}\right)$, $\lambda_{NL}^{si}=\theta_{NL}^{si}\left(1+\frac{\rho_{NL}}{\sqrt{k^{N}k^{L}}}\right)$

represent the aggregation-corrected cofeeding transmission rates on recovered (*r*) or susceptible/infected (*si*) hosts. LL = from a larva to a larva, LN = from a nymph to a larva, NN = from a nymph to a nymph, NL = from a larva to a nymph.

and,

$P_{H1}^{Z}=$Proportion of H_1_ hosts encountered = $\frac{\beta_{1}^{Z}H_{1}}{\beta_{1}^{Z}H_{1}+\beta_{2}^{Z}H_{2}}$

$P_{H2}^{Z}=$Proportion of H_2_ hosts encountered = $\frac{\beta_{2}^{Z}H_{2}}{\beta_{1}^{Z}H_{1}+\beta_{2}^{Z}H_{2}}$

$P_{H1^{r}}^{Z}=$Proportion of $H_{1}^{r}$hosts encountered = $\frac{\beta_{1}^{Z}H_{1}^{r}}{\beta_{1}^{Z}H_{1}+\beta_{2}^{Z}H_{2}}$

$P_{H1^{s}+H1^{i}}^{Z}=$Proportion of $H_{1}^{s}$ or $H_{1}^{i}$ hosts encountered = $\frac{\beta_{1}^{Z}\left(H_{1}^{s}+H_{1}^{i}\right)}{\beta_{1}^{Z}H_{1}+\beta_{2}^{Z}H_{2}}$

$P_{H1^{i}}^{Z}=$Proportion of $H_{1}^{i}$ hosts encountered = $\frac{\beta_{1}^{Z}H_{1}^{i}}{\beta_{1}^{Z}H_{1}+\beta_{2}^{Z}H_{2}}$

$P_{H1^{s}+H1^{r}}^{Z}=$Proportion of $H_{1}^{s}$ or $H_{1}^{r}$ hosts encountered = $\frac{\beta_{1}^{Z}\left(H_{1}^{s}+H_{1}^{r}\right)}{\beta_{1}^{Z}H_{1}+\beta_{2}^{Z}H_{2}}$

Then, the rate of cofeeding transmission of larvae

$$\begin{matrix} =P_{LL}+P_{LN}\left(1-P_{LL}\right)=P_{LN}+P_{LL}\left(1-P_{LN}\right) \\ =\left[1-\exp\left(-\frac{L_{Q,t}^{i}P_{f}^{L}\left(\lambda_{LL}^{r}P_{H1^{r}}^{L}+\lambda_{LL}^{si}P_{H1^{s}+H1^{i}}^{L}\right)}{H_{1}}\right)\right] \\ +\left[1-\exp\left(-\frac{N_{Q,t}^{i}P_{f}^{N}\left(\lambda_{LN}^{r}P_{H1^{r}}^{N}+\lambda_{LN}^{si}P_{H1^{s}+H1^{i}}^{N}\right)}{H_{1}}\right)\right] \\ \cdot\exp\left(-\frac{L_{Q,t}^{i}P_{f}^{L}\left(\lambda_{LL}^{r}P_{H1^{r}}^{L}+\lambda_{LL}^{si}P_{H1^{s}+H1^{i}}^{L}\right)}{H_{1}}\right) \end{matrix}$$

That of nymphs

$$\begin{matrix} =P_{NN}+P_{NL}\left(1-P_{NN}\right)=P_{NL}+P_{NN}\left(1-P_{NL}\right) \\ =\left[1-\exp\left(-\frac{N_{Q,t}^{i}P_{f}^{N}\left(\lambda_{NN}^{r}P_{H1^{r}}^{N}+\lambda_{NN}^{si}P_{H1^{s}+H1^{i}}^{N}\right)}{H_{1}}\right)\right] \\ +\left[1-\exp\left(-\frac{L_{Q,t}^{i}P_{f}^{L}\left(\lambda_{NL}^{r}P_{H1^{r}}^{L}+\lambda_{NL}^{si}P_{H1^{s}+H1^{i}}^{L}\right)}{H_{1}}\right)\right] \\ \cdot\exp\left(-\frac{N_{Q,t}^{i}P_{f}^{N}\left(\lambda_{NN}^{r}P_{H1^{r}}^{N}+\lambda_{NN}^{si}P_{H1^{s}+H1^{i}}^{N}\right)}{H_{1}}\right) \end{matrix}$$

These rates are zero for adult ticks.

The monthly equations of the ticks (with cohort overlap)

July

$$L_{U,t+1}^{s}=a_{T}\cdot viab\cdot\left(A_{F,t}^{s}\cdot\exp\left(-d_{F}^{A}\right)\right)+\left(1-\varepsilon^{0}\right)a_{T}\cdot viab\cdot\left(A_{F,t}^{i}\cdot\exp\left(-d_{F}^{A}\right)\right)$$

$$L_{F,t+1}^{s}=0$$

$$L_{U,t+1}^{i}=a_{T}\cdot viab\cdot\varepsilon^{0}\left(A_{F,t}^{i}\cdot\exp\left(-d_{F}^{A}\right)\right)$$

$$L_{F,t+1}^{i}=0$$

$$N_{U,t+1}^{s}=N_{U,t}^{s}\cdot\left(1-P_{f}^{N}\right)\cdot\exp\left(-d_{U}^{N}\right)$$

$$N_{F,t+1}^{s}=N_{U,t}^{s}\cdot\exp\left(-d_{U}^{N}\right)\cdot FEED\_NOT\_INFECTED^{N}+N_{F,t}^{s}\cdot\exp\left(-d_{F}^{N}\right)$$

$$N_{U,t+1}^{i}=N_{U,t}^{i}\cdot\left(1-P_{f}^{N}\right)\cdot\exp\left(-d_{U}^{N}\right)$$

$$\begin{matrix} N_{F,t+1}^{i}=N_{U,t}^{i}\cdot P_{f}^{N}\cdot\exp\left(-d_{U}^{N}\right)+N_{U,t}^{s}\cdot FEED\_AND\_INFECTED^{N}\cdot\exp\left(-d_{U}^{N}\right) \\ +N_{F,t}^{i}\cdot\exp\left(-d_{F}^{N}\right) \end{matrix}$$

$$A_{t+1}=0$$

August

$$L_{U,t+1}^{s}=L_{U,t}^{s}\cdot\left(1-P_{f}^{L}\right)\cdot\exp\left(-d_{U}^{L}\right)$$

$$L_{F,t+1}^{s}=L_{U,t}^{s}\cdot\exp\left(-d_{U}^{L}\right)\cdot FEED\_NOT\_INFECTED^{L}$$

$$L_{U,t+1}^{i}=L_{U,t}^{i}\cdot\left(1-P_{f}^{L}\right)\cdot\exp\left(-d_{U}^{L}\right)$$

$$L_{F,t+1}^{i}=L_{U,t}^{i}\cdot P_{f}^{L}\cdot\exp\left(-d_{U}^{L}\right)+L_{U,t}^{s}\cdot FEED\_AND\_INFECTED^{L}\cdot\exp\left(-d_{U}^{L}\right)$$

$$N_{U,t+1}^{s}=N_{U,t}^{s}\cdot\left(1-P_{f}^{N}\right)\cdot\exp\left(-d_{U}^{N}\right)$$

$$N_{F,t+1}^{s}=N_{U,t}^{s}\cdot\exp\left(-d_{U}^{N}\right)\cdot FEED\_NOT\_INFECTED^{N}+N_{F,t}^{s}\cdot\exp\left(-d_{F}^{N}\right)$$

$$N_{U,t+1}^{i}=N_{U,t}^{i}\cdot\left(1-P_{f}^{N}\right)\cdot\exp\left(-d_{U}^{N}\right)$$

$$\begin{matrix} N_{F,t+1}^{i}=N_{U,t}^{i}\cdot P_{f}^{N}\cdot\exp\left(-d_{U}^{N}\right)+N_{U,t}^{s}\cdot FEED\_AND\_INFECTED^{N} \\ \cdot\exp\left(-d_{U}^{N}\right)+N_{F,t}^{i}\cdot\exp\left(-d_{F}^{N}\right) \end{matrix}$$

$$A_{t+1}=0$$

September

$$L_{U,t+1}^{s}=L_{U,t}^{s}\cdot\left(1-P_{f}^{L}\right)\cdot\exp\left(-d_{U}^{L}\right)$$

$$L_{F,t+1}^{s}=L_{U,t}^{s}\cdot\exp\left(-d_{U}^{L}\right)\cdot FEED\_NOT\_INFECTED^{L}+L_{F,t}^{s}\cdot\exp\left(-d_{F}^{L}\right)$$

$$L_{U,t+1}^{i}=L_{U,t}^{i}\cdot\left(1-P_{f}^{L}\right)\cdot\exp\left(-d_{U}^{L}\right)$$

$$\begin{matrix} L_{F,t+1}^{i}=L_{U,t}^{i}\cdot P_{f}^{L}\cdot\exp\left(-d_{U}^{L}\right)+L_{U,t}^{s}\cdot FEED\_AND\_INFECTED^{L}\cdot\exp\left(-d_{U}^{L}\right) \\ +L_{F,t}^{i}\cdot\exp\left(-d_{F}^{L}\right) \end{matrix}$$

$$N_{Q,t+1}^{s}=0$$

$$N_{F,t+1}^{s}=N_{U,t}^{s}\cdot\exp\left(-d_{U}^{N}\right)\cdot FEED\_NOT\_INFECTED^{N}+N_{F,t}^{s}\cdot\exp\left(-d_{F}^{N}\right)$$

$$N_{Q,t+1}^{i}=0$$

$$\begin{matrix} N_{F,t+1}^{i}=N_{U,t}^{i}\cdot P_{f}^{N}\cdot\exp\left(-d_{U}^{N}\right)+N_{U,t}^{s}\cdot FEED\_AND\_INFECTED^{N}\cdot\exp\left(-d_{U}^{N}\right) \\ +N_{F,t}^{i}\cdot\exp\left(-d_{F}^{N}\right) \end{matrix}$$

$$A_{t+1}=0$$

October

$$L_{U,t+1}^{s}=L_{U,t}^{s}\cdot\left(1-P_{f}^{L}\right)\cdot\exp\left(-d_{U}^{L}\right)$$

$$L_{F,t+1}^{s}=L_{U,t}^{s}\cdot\exp\left(-d_{U}^{L}\right)\cdot FEED\_NOT\_INFECTED^{L}+L_{F,t}^{s}\cdot\exp\left(-d_{F}^{L}\right)$$

$$L_{U,t+1}^{i}=L_{U,t}^{i}\cdot\left(1-P_{f}^{L}\right)\cdot\exp\left(-d_{U}^{L}\right)$$

$$\begin{matrix} L_{F,t+1}^{i}=L_{U,t}^{i}\cdot P_{f}^{L}\cdot\exp\left(-d_{U}^{L}\right)+L_{U,t}^{s}\cdot FEED\_AND\_INFECTED^{L}\cdot\exp\left(-d_{U}^{L}\right) \\ +L_{F,t}^{i}\cdot\exp\left(-d_{F}^{L}\right) \end{matrix}$$

$$N_{t+1}=0$$

$$A_{U,t+1}^{s}=m^{N}\cdot\left(N_{F,t}^{s}\cdot\exp\left(-d_{F}^{N}\right)\right)+\left(1-\varepsilon^{N}\right)\cdot m^{N}\cdot\left(N_{F,t}^{i}\cdot\exp\left(-d_{F}^{N}\right)\right)$$

$$A_{F,t+1}^{s}=0$$

$$A_{U,t+1}^{i}=\varepsilon^{N}\cdot m^{N}\cdot\left(N_{F,t}^{i}\cdot\exp\left(-d_{F}^{N}\right)\right)$$

$$A_{F,t+1}^{i}=0$$

November

$$L_{U,t+1}^{s}=0$$

$$L_{F,t+1}^{s}=L_{U,t}^{s}\cdot\exp\left(-d_{U}^{L}\right)\cdot FEED\_NOT\_INFECTED^{L}+L_{F,t}^{s}\cdot\exp\left(-d_{F}^{L}\right)$$

$$L_{U,t+1}^{i}=0$$

$$\begin{matrix} L_{F,t+1}^{i}=L_{U,t}^{i}\cdot P_{f}^{L}\cdot\exp\left(-d_{U}^{L}\right)+L_{U,t}^{s}\cdot FEED\_AND\_INFECTED^{L}\cdot\exp\left(-d_{U}^{L}\right) \\ +L_{F,t}^{i}\cdot\exp\left(-d_{F}^{L}\right) \end{matrix}$$

$$N_{t+1}=0$$

$$A_{U,t+1}^{s}=A_{U,t}^{s}\cdot\left(1-P_{f}^{A}\right)\cdot\exp\left(-d_{U}^{A}\right)$$

$$A_{F,t+1}^{s}=A_{U,t}^{s}\cdot\exp\left(-d_{U}^{A}\right)\cdot FEED\_NOT\_INFECTED^{A}$$

$$A_{U,t+1}^{i}=A_{U,t}^{i}\cdot\left(1-P_{f}^{A}\right)\cdot\exp\left(-d_{U}^{A}\right)$$

$$A_{F,t+1}^{i}=A_{U,t}^{i}\cdot P_{f}^{A}\cdot\exp\left(-d_{U}^{A}\right)+A_{U,t}^{s}\cdot FEED\_AND\_INFECTED^{A}\cdot\exp\left(-d_{U}^{A}\right)$$

December

$$L_{t+1}=0$$

$$N_{U,t+1}^{s}=m^{L}\cdot L_{F,t}^{s}\cdot\exp\left(-d_{F}^{L}\right)+\left(1-\varepsilon^{L}\right)\cdot m^{L}\cdot L_{F,t}^{i}\cdot\exp\left(-d_{F}^{L}\right)$$

$$N_{F,t+1}^{s}=0$$

$$N_{U,t+1}^{i}=\varepsilon^{L}\cdot m^{L}\cdot L_{F,t}^{i}\cdot\exp\left(-d_{U}^{L}\right)$$

$$N_{F,t+1}^{i}=0$$

$$A_{U,t+1}^{s}=A_{U,t}^{s}\cdot\left(1-P_{f}^{A}\right)\cdot\exp\left(-d_{U}^{A}\right)$$

$$A_{F,t+1}^{s}=A_{U,t}^{s}\cdot\exp\left(-d_{U}^{A}\right)\cdot FEED\_NOT\_INFECTED^{A}+A_{F,t}^{s}\cdot\exp\left(-d_{F}^{A}\right)$$

$$A_{U,t+1}^{i}=A_{U,t}^{i}\cdot\left(1-P_{f}^{A}\right)\cdot\exp\left(-d_{U}^{A}\right)$$

$$\begin{matrix} A_{F,t+1}^{i}=A_{U,t}^{i}\cdot P_{f}^{A}\cdot\exp\left(-d_{U}^{A}\right)+A_{U,t}^{s}\cdot FEED\_AND\_INFECTED^{A}\cdot\exp\left(-d_{U}^{A}\right) \\ +A_{F,t}^{i}\cdot\exp\left(-d_{F}^{A}\right) \end{matrix}$$

January

$$L_{t+1}=0$$

$$N_{U,t+1}^{s}=N_{U,t}^{s}\cdot\exp\left(-d_{U}^{N}\right)$$

$$N_{F,t+1}^{s}=0$$

$$N_{U,t+1}^{i}=N_{U,t}^{i}\cdot\exp\left(-d_{U}^{N}\right)$$

$$N_{F,t+1}^{i}=0$$

$$A_{U,t+1}^{s}=A_{U,t}^{s}\cdot\left(1-P_{f}^{A}\right)\cdot\exp\left(-d_{U}^{A}\right)$$

$$A_{F,t+1}^{s}=A_{U,t}^{s}\cdot\exp\left(-d_{U}^{A}\right)\cdot FEED\_NOT\_INFECTED^{A}+A_{F,t}^{s}\cdot\exp\left(-d_{F}^{A}\right)$$

$$A_{U,t+1}^{i}=A_{U,t}^{i}\cdot\left(1-P_{f}^{A}\right)\cdot\exp\left(-d_{U}^{A}\right)$$

$$\begin{matrix} A_{F,t+1}^{i}=A_{U,t}^{i}\cdot P_{f}^{A}\cdot\exp\left(-d_{U}^{A}\right)+A_{U,t}^{s}\cdot FEED\_AND\_INFECTED^{A}\cdot\exp\left(-d_{U}^{A}\right) \\ +A_{F,t}^{i}\cdot\exp\left(-d_{F}^{A}\right) \end{matrix}$$

February

$$L_{t+1}=0$$

$$N_{U,t+1}^{s}=N_{U,t}^{s}\cdot\exp\left(-d_{U}^{N}\right)$$

$$N_{F,t+1}^{s}=0$$

$$N_{U,t+1}^{i}=N_{U,t}^{i}\cdot\exp\left(-d_{U}^{N}\right)$$

$$N_{F,t+1}^{i}=0$$

$$A_{U,t+1}^{s}=A_{U,t}^{s}\cdot\exp\left(-d_{U}^{A}\right)$$

$$A_{F,t+1}^{s}=A_{F,t}^{s}\cdot\exp\left(-d_{F}^{A}\right)$$

$$A_{U,t+1}^{i}=A_{U,t}^{i}\cdot\exp\left(-d_{U}^{A}\right)$$

$$A_{F,t+1}^{i}=A_{F,t}^{i}\cdot\exp\left(-d_{F}^{A}\right)$$

March

$$L_{t+1}=0$$

$$N_{U,t+1}^{s}=N_{U,t}^{s}\cdot\exp\left(-d_{U}^{N}\right)$$

$$N_{F,t+1}^{s}=0$$

$$N_{U,t+1}^{i}=N_{U,t}^{i}\cdot\exp\left(-d_{U}^{N}\right)$$

$$N_{F,t+1}^{i}=0$$

$$A_{U,t+1}^{s}=A_{U,t}^{s}\cdot\exp\left(-d_{U}^{A}\right)$$

$$A_{F,t+1}^{s}=A_{F,t}^{s}\cdot\exp\left(-d_{F}^{A}\right)$$

$$A_{U,t+1}^{i}=A_{U,t}^{i}\cdot\exp\left(-d_{U}^{A}\right)$$

$$A_{F,t+1}^{i}=A_{F,t}^{i}\cdot\exp\left(-d_{F}^{A}\right)$$

April

$$L_{t+1}=0$$

$$N_{U,t+1}^{s}=N_{U,t}^{s}\cdot\exp\left(-d_{U}^{N}\right)$$

$$N_{F,t+1}^{s}=0$$

$$N_{U,t+1}^{i}=N_{U,t}^{i}\cdot\exp\left(-d_{U}^{N}\right)$$

$$N_{F,t+1}^{i}=0$$

$$A_{U,t+1}^{s}=A_{U,t}^{s}\cdot\left(1-P_{f}^{A}\right)\cdot\exp\left(-d_{U}^{A}\right)$$

$$A_{F,t+1}^{s}=A_{U,t}^{s}\cdot\exp\left(-d_{U}^{A}\right)\cdot FEED\_NOT\_INFECTED^{A}+A_{F,t}^{s}\cdot\exp\left(-d_{F}^{A}\right)$$

$$A_{U,t+1}^{i}=A_{U,t}^{i}\cdot\left(1-P_{f}^{A}\right)\cdot\exp\left(-d_{U}^{A}\right)$$

$$\begin{matrix} A_{F,t+1}^{i}=A_{U,t}^{i}\cdot P_{f}^{A}\cdot\exp\left(-d_{U}^{A}\right)+A_{U,t}^{s}\cdot FEED\_AND\_INFECTED^{A}\cdot\exp\left(-d_{U}^{A}\right) \\ +A_{F,t}^{i}\cdot\exp\left(-d_{F}^{A}\right) \end{matrix}$$

May

$$L_{t+1}=0$$

$$N_{U,t+1}^{s}=N_{U,t}^{s}\cdot\exp\left(-d_{U}^{N}\right)$$

$$N_{F,t+1}^{s}=0$$

$$N_{U,t+1}^{i}=N_{U,t}^{i}\cdot\exp\left(-d_{U}^{N}\right)$$

$$N_{F,t+1}^{i}=0$$

$$A_{U,t+1}^{s}=A_{U,t}^{s}\cdot\left(1-P_{f}^{A}\right)\cdot\exp\left(-d_{U}^{A}\right)$$

$$A_{F,t+1}^{s}=A_{U,t}^{s}\cdot\exp\left(-d_{U}^{A}\right)\cdot FEED\_NOT\_INFECTED^{A}+A_{F,t}^{s}\cdot\exp\left(-d_{F}^{A}\right)$$

$$A_{U,t+1}^{i}=A_{U,t}^{i}\cdot\left(1-P_{f}^{A}\right)\cdot\exp\left(-d_{U}^{A}\right)$$

$$\begin{matrix} A_{F,t+1}^{i}=A_{U,t}^{i}\cdot P_{f}^{A}\cdot\exp\left(-d_{U}^{A}\right)+A_{U,t}^{s}\cdot FEED\_AND\_INFECTED^{A}\cdot\exp\left(-d_{U}^{A}\right) \\ +A_{F,t}^{i}\cdot\exp\left(-d_{F}^{A}\right) \end{matrix}$$

June

$$L_{t+1}=0$$

$$N_{U,t+1}^{s}=N_{U,t}^{s}\cdot\left(1-P_{f}^{N}\right)\cdot\exp\left(-d_{U}^{N}\right)$$

$$N_{F,t+1}^{s}=N_{U,t}^{s}\cdot\exp\left(-d_{U}^{N}\right)\cdot FEED\_NOT\_INFECTED^{N}$$

$$N_{U,t+1}^{i}=N_{U,t}^{i}\cdot\left(1-P_{f}^{N}\right)\cdot\exp\left(-d_{U}^{N}\right)$$

$$N_{F,t+1}^{i}=N_{U,t}^{i}\cdot P_{f}^{N}\cdot\exp\left(-d_{U}^{N}\right)+N_{U,t}^{s}\cdot FEED\_AND\_INFECTED^{ N}\cdot\exp\left(-d_{U}^{N}\right)$$

$$A_{U,t+1}^{s}=0$$

$$A_{F,t+1}^{s}=A_{U,t}^{s}\cdot\exp\left(-d_{U}^{A}\right)\cdot FEED\_NOT\_INFECTED^{A}+A_{F,t}^{s}\cdot\exp\left(-d_{F}^{A}\right)$$

$$A_{U,t+1}^{i}=0$$

$$\begin{matrix} A_{F,t+1}^{i}=A_{U,t}^{i}\cdot P_{f}^{A}\cdot\exp\left(-d_{U}^{A}\right)+A_{U,t}^{s}\cdot FEED\_AND\_INFECTED^{A}\cdot\exp\left(-d_{U}^{A}\right) \\ +A_{F,t}^{i}\cdot\exp\left(-d_{F}^{A}\right) \end{matrix}$$

The host equations

To accommodate the time scales of the host infectious period (2 – 3 days), we approximate the monthly host dynamics using discrete-time equations derived from the integration of differential equations that describe the within-month infection, recovery, and mortality processes in the host population.

$$\begin{matrix} H_{\tau+1}^{s}=H_{\tau}^{s}\exp\left[-\left(\lambda_{\tau}+d^{H}\right)\right] \\ H_{\tau+1}^{i}=H_{\tau}^{i}\exp\left[-\left(\gamma+d^{H}\right)\right]+\frac{\lambda_{\tau}H_{\tau}^{s}}{\gamma-\lambda_{\tau}}\left[\exp\left(-\lambda_{\tau}\right)-\exp\left(-\gamma\right)\right]\exp\left(-d^{H}\right) \\ H_{\tau+1}^{r}=H_{\tau}^{r}\exp\left(-d^{H}\right)+H_{\tau}^{i}\left[1-\exp\left(-\gamma\right)\right]\exp\left(-d^{H}\right)+H_{\tau}^{s}\left[1-\frac{\gamma}{\gamma-\lambda_{\tau}}\exp\left(-\lambda_{\tau}\right)+\frac{\lambda_{\tau}}{\gamma-\lambda_{\tau}}\exp\left(-\gamma\right)\right]\exp\left(-d^{H}\right) \end{matrix}$$

**References**

1. Rosà R, Pugliese A. (2007) Effects of tick population dynamics and host densities on the persistence of tick-borne infections. Mathematical BioSciences 208: 216–240.
2. Labuda M, Kozuch O, Zuffova E, Eleckova E, Hails R et al. (1997) Tick-borne encephalitis virus transmission between ticks cofeeding on specific immune natural rodent hosts. Virology 235: 138-143.
3. Randolph SE, Miklisova D, Lysy J, Rogers DJ, Labuda M. (1999) Incidence from coincidence: patterns of tick infestations on rodents facilitate transmission of tick-borne encephalitis virus. Parasitology 118: 177-186.
4. Brunner JL, Ostfeld RS. (2008) Multiple causes of variable tick burdens on small-mammal hosts. Ecology 89: 2259-2272.
5. Rosà R, Pugliese A, Norman R, Hudson PJ. (2003) Thresholds for disease persistence in models for tick-borne infections including non-viraemic transmission, extended feeding and tick aggregation. Journal of Theoretical Biology 224: 359-376.
6. Lackey JA, Huckaby DG, Ormiston BG. (1985) *Peromyscus leucopus.* In Mammalian species, vol. 247, pp. 1-10.
